# Metabolic profiles of cutaneous lupus have abnormalities in the nicotinamide adenine dinucleotide pathway

**DOI:** 10.1136/lupus-2024-001401

**Published:** 2025-01-25

**Authors:** Laila F Abbas, Grant Barber, Grace Lu, Bahir Chamseddin, Hieu Vu, Ling Cai, Divya Srivastava, Rajiv L Nijhawan, Richard C Wang, Benjamin F Chong

**Affiliations:** 1 Dermatology, The University of Texas Southwestern Medical Center, Dallas, Texas, USA; 2 Children’s Medical Center Research Institute, University of Texas Southwestern, Dallas, Texas, USA; 3 Population and Data Sciences, University of Texas Southwestern, Dallas, Texas, USA

**Keywords:** Autoimmune Diseases, Lupus Erythematosus, Systemic, Therapeutics

## Abstract

**Objective:**

Metabolic reprogramming plays a critical role in modulating the innate and adaptive immune response, but its role in cutaneous autoimmune diseases, such as cutaneous lupus erythematosus (CLE), is less well studied. An improved understanding of the metabolic pathways dysregulated in CLE may lead to novel treatment options, biomarkers and insights into disease pathogenesis. The objective was to compare metabolomic profiles in the skin and sera of CLE and control patients using liquid chromatography–mass spectrometry (LC-MS).

**Methods:**

This was a cross-sectional pilot study comparing metabolomic sera and skin profiles of patients with CLE and normal controls. Patients were recruited from outpatient dermatology clinics at the University of Texas Southwestern and Parkland Health in Dallas, Texas, from January 2019 to October 2020. Skin and serum samples underwent LC-MS analysis. Disease sample metabolite levels were compared with controls, with significance levels adjusted for multiple hypothesis testing.

**Results:**

17 serum samples (9 CLE, 8 control) and 11 skin samples (5 CLE, 6 control) were analysed using LC-MS, yielding 313 known unique metabolic structures from CLE samples. Patients with CLE were found to have 11 metabolites of differential abundance in the skin, but only 2 in the sera. CLE skin showed increased levels of citrulline (log_2_ fold change (FC)=1.15, p=0.02) and uracil (log_2_FC=1.79, p=0.04), and downregulation of cyclic ADP ribose (cADPr) (log_2_FC=0.83, p=0.04), nicotinamide mononucleotide (NMN) (log_2_FC=0.75, p=0.016) and nicotinamide adenine dinucleotide (NAD^+^) (log_2_FC=0.86, p=0.016) versus control skin. CLE sera had increased arabinose (log_2_FC=1.17, p=0.02) and cystine (log_2_FC=1.04, p=0.03) compared with control sera.

**Conclusions:**

Metabolites associated with the NAD^+^ pathway may be dysregulated in the skin of patients with CLE. Available treatments including nicotinamide supplementation and anti-CD38 biologics that can correct these abnormalities can be further investigated in patients with CLE.

**WHAT IS ALREADY KNOWN ON THIS TOPIC**

- Cutaneous lupus erythematosus (CLE) is an autoimmune disease whose pathophysiology is incompletely understood.
- Metabolomics has been used to identify novel diagnostic and therapeutic targets in genetic diseases and malignancies.
- Metabolic reprogramming plays a critical role in modulating the innate and adaptive immune response, but its role in autoimmune skin diseases, such as CLE, is less well studied.

**WHAT THIS STUDY ADDS**

- An unbiased liquid chromatography–mass spectrometry (LC-MS) metabolomics approach was used to compare metabolic profiles of CLE and control skin and serum.
- CLE versus control skin samples showed an increased number of metabolites with differential abundance compared with CLE versus control sera.
- Several metabolites related to the nicotinamide adenine dinucleotide (NAD^+^) pathway showed significantly decreased abundance in CLE skin.

**HOW THIS STUDY MIGHT AFFECT RESEARCH, PRACTICE OR POLICY**

- An improved understanding of the metabolic pathways involved in CLE may lead to novel treatment options, biomarker discovery and insight into disease pathogenesis.
- This pilot study revealed unique metabolite profiles in the skin and serum of patients with CLE and identified abnormalities in NAD^+^ metabolism, which may have therapeutic implications.

## Introduction

 Cutaneous lupus erythematosus (CLE) is a autoimmune skin disorder that can occur in the presence or absence of SLE. While both genetic and environmental factors contribute to the development of CLE, the precise pathophysiology of CLE remains incompletely understood.

Metabolomic profiling has emerged as an approach to bridge genetic information to dysfunctional and clinically relevant pathways. Metabolomics, the comprehensive analysis of metabolites in a specimen, may allow for a more detailed characterisation of disease phenotypes, an understanding of metabolic derangements that underlie disease and identification of new therapeutic targets. Metabolic reprogramming plays a critical role in modulating the innate and adaptive immune response, but its role in specific inflammatory diseases, especially CLE, is less well-studied.^1^

The objective of this study was to perform an unbiased metabolomics analysis of both skin and sera of patients with CLE and compare them to controls. We hypothesised that the metabolites of CLE skin and sera would differ from those of controls and uncover metabolic pathways for novel therapies in CLE.

## Methods

### Study design

In this cross-sectional study, we examined the metabolite profiles of sera and skin of patients with CLE and controls using liquid chromatography–mass spectrometry (LC-MS). Patients with CLE and controls were recruited from the University of Texas Southwestern Medical Center and Parkland Health outpatient dermatology clinics seen from January 2019 to October 2020. Inclusion criteria included those with a diagnosis of CLE confirmed by a dermatologist (BFC) with clinicopathological correlation, and with skin or serum samples collected within 1 year of metabolite analysis. Exclusion criteria included patients with SLE and age <18 years. Blood serum samples and/or skin biopsies were collected at their visit, primarily while patients were not on systemic therapy to avoid medication effects on metabolomic analyses. Three participants with CLE contributed both skin and sera to this study. Control sera were obtained from patients without active skin disease or history of autoimmunity, and control skin samples were obtained from dog-ear skin from Mohs surgery patients without history of autoimmune diseases. Demographic and clinical information, including current medications and skin disease severity scores (ie, Cutaneous Lupus Erythematosus Disease Area and Severity Index (CLASI)), were collected. All participants provided written, informed consent.

### Metabolomics assay

Unbiased LC-MS metabolomics was used to characterise the skin and sera of patients with CLE. Patient serum and skin samples were processed and stored and further described in online supplemental methods. A SpeedVac was used to dry the supernatant samples without heat to produce a dried pellet of the metabolites. Data acquisition was performed by reverse-phase chromatography on a 1290 ultra-high performance LC system interfaced to 6550 iFunnel Q-TOF high-resolution mass spectrometer (Agilent Technologies, California, USA). The MS was operated in both positive and negative (ESI+ and ESI-) modes. Analytes were separated on an Acquity UPLC HSS T3 column (1.8 µm, 2.1×150 mm, Waters, Massachusetts, USA). Raw data files were processed using Profinder B.08.00 SP3 software (Agilent Technologies) with an in-house database containing retention time and accurate mass information on 600 standards from Mass Spectrometry Metabolite Library (IROA Technologies, Massachusetts, USA), which was created under the same analysis conditions. The in-house database matching parameters were mass tolerance of 10 ppm and retention time tolerance of 0.5 min. Peak integration result was manually curated in Profinder for improved consistency.

### Metabolomics data analysis

Preliminary statistical models included principal component analysis (PCA) and heatmaps. PCA and heatmaps were first used to visualise the distribution of control and CLE samples for the evaluation of sera and skin metabolic profiling. Statistical analysis such as unpaired t-tests were then implemented on log2 transformed metabolite values to obtain the discriminating metabolites. Significance levels were adjusted for multiple hypothesis testing according to Benjamini and Hochberg, with p values <0.05 considered significant. Spearman correlation analyses were performed to examine relationships between skin and sera metabolites of interest versus CLASI activity (CLASI-A) scores. All statistical analysis was performed using R and GraphPad Prism V.10.

## Results

**Figure 1:**
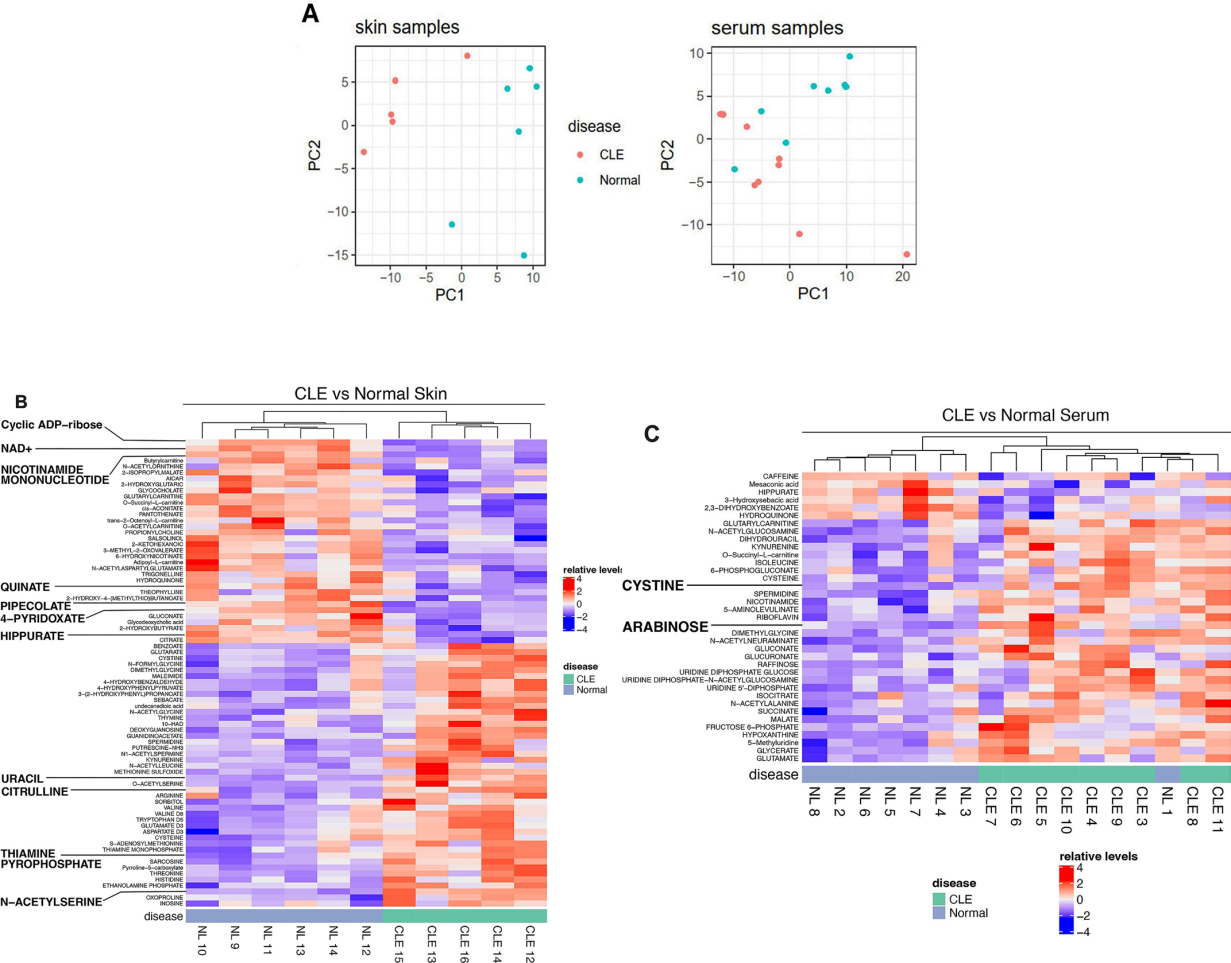
Metabolite profiles of cutaneous lupus erythematosus (CLE) sera (n=9) versus control sera (n=8) and CLE skin (n=5) versus control skin (n=6) shown through principal component analysis (PCA) plot (A) and heatmaps (B, C). (A) PCA plots demonstrate differential clustering of CLE sera compared with control sera and CLE skin compared with control skin, with distinct clustering notable in skin samples. (B, C) Eleven skin metabolites and two sera metabolites were significantly different in CLE and normal samples. The heatmaps for skin (B) and sera (C) contain these differentially expressed metabolites highlighted by larger text. Red and blue represents higher and lower expression, respectively. NAD+, nicotinamide adenine dinucleotide.

Patient demographic and clinical information of CLE and controls are summarised in online supplemental table 1. From a library of 313 known unique metabolites, differences in metabolite expression were calculated in five CLE skin versus six control skin samples (online supplemental table 2) and nine CLE sera versus eight control sera (online supplemental table 3). PCA plots showed more pronounced differences in CLE and normal skin compared with CLE and normal sera (figure 1A). Compared with normal controls, patients with CLE had 11 metabolites of differential abundance in the skin and two in the serum. Heatmaps of metabolite expression of CLE and normal skin (figure 1B) and sera (figure 1C) were generated. Significant differences in sera included increased arabinose (log_2_ fold change (FC): 1.17, p=0.02) and cystine (log_2_FC: 1.04, p=0.03) in CLE versus control sera. Key changes in metabolites in CLE skin included the upregulation of citrulline (log_2_FC: 1.15, p=0.03) and uracil (log_2_FC: 1.18, p=0.04), and downregulation of cyclic-ADP ribose (cADPr) (log_2_FC: 0.83; p=0.04), nicotinamide mononucleotide (NMN) (log_2_FC: 0.76; p=0.02) and nicotinamide adenine dinucleotide (NAD^+^) (log_2_FC: 0.86; p=0.02) (table 1). Spearman correlation analyses between CLASI-A scores and metabolites of interest revealed a significant correlation for serum arabinose (ρ=0.7, p=0.04) (online supplemental table 4).

**Table 1:** Differentially expressed metabolites in CLE versus control skin and CLE versus control sera

Compound	Serum or skin	CLE mean peak intensity	Control mean peak intensity	Log_2_ Fold change	P value
Cystine	Serum	13.65	13.08	1.04	0.03
Arabinose	Serum	12.82	10.99	1.17	0.02
Citrulline	Skin	21.49	18.74	1.15	0.03
Uracil	Skin	13.43	11.37	1.18	0.04
*N*-acetylserine	Skin	14.62	12.88	1.14	0.02
Thiamine pyrophosphate	Skin	13.21	11.09	1.19	0.05
cADPr	Skin	16.30	19.70	0.83	0.04
NMN	Skin	14.67	19.36	0.76	0.02
NAD^+^	Skin	20.41	23.61	0.86	0.02
4-Pyridoxate	Skin	8.31	10.24	0.81	0.006
Hippurate	Skin	11.48	15.54	0.74	0.02
Pipecolate	Skin	17.12	18.47	0.93	0.02
Quinate	Skin	11.29	14.60	0.77	0.04

cADPr, cyclic-ADP ribose; CLE, cutaneous lupus erythematosus; NAD, nicotinamide adenine dinucleotide; NMN, nicotinamide mononucleotide

## Discussion

In this unbiased metabolomics study, our results indicated differences in metabolites that distinguish CLE sera from control sera and CLE skin from control skin. Notably, our novel approach comparing metabolites in CLE and control skin and sera demonstrated that CLE skin showed increased uniquely expressed metabolites versus control skin compared with CLE versus control sera. This emphasises the important contribution of the skin to the pathology of CLE. The exclusion of patients with SLE, which can often present with CLE, may have contributed to the relatively fewer metabolite differences between CLE and control sera.

**Figure 2:**
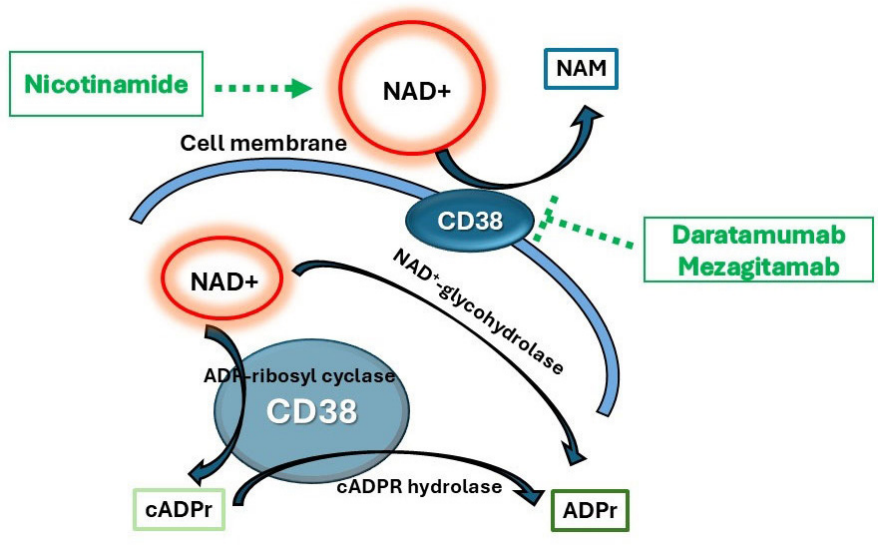
CD38 in the nicotinamide adenine dinucleotide (NAD^+^) salvage pathway. At the cell membrane, CD38 is a NADase that catabolyses NAD^+^ into nicotinamide (NAM). On the membranes of cellular organelles such as mitochondria, CD38 has multiple enzymatic activities by converting NAD^+^ into cyclic ADP ribose (cADPr) as a cyclase or ADPr as a glycohydrolase, and hydrolyses cADPr to ADPr as a hydrolase. We hypothesise that increased CD38 activity in lupus can lead to decreased NAD^+^ and cADPr levels in cutaneous lupus erythematosus (CLE) skin, as shown in the metabolomics assay. Potential treatment strategies for CLE include NAD^+^ supplementation through nicotinamide and CD38 antibody blockade via daratamumab and mezagitamab (labelled in green).

Numerous metabolites were found to be differentially expressed in CLE skin samples compared with controls. Of note, cADPr, NAD^+^ and NMN were downregulated in CLE skin compared with controls. These metabolites are involved in the salvage pathway of NAD^+^ metabolism. The multifunctional CD38 enzyme catabolyses NAD^+^ into cADPr and ADP ribose (ADPr), and hydrolyses cADPr to ADPr (figure 2).^2^ Moreover, CD38 can also hydrolyse NMN in vivo.^3^ Dysregulation of the CD38/NAD axis has been implicated in the pathogenesis of numerous rheumatic diseases including systemic sclerosis, SLE and rheumatoid arthritis.^4 5^ Hyperactivation of CD38 promotes chronic inflammation, likely through a B-cell-dependent mechanism.^6^ CD38 has been identified as a therapeutic target for refractory SLE. Daratamumab, an United States Food and Drug Administration (FDA) approved anti-CD38 biologic for multiple myeloma, was trialled in two patients with life-threatening SLE, who clinically improved. It caused depletion of plasma cells, reduction of type I interferon (IFN) activity, decreased gene expression associated with T cell activation in those patients.^7^ Another anti-CD38 mAb, mezagitamab, was investigated in a phase 1B, double-blind, placebo-controlled study of 22 patients with SLE. While there were no differences in disease activity indices in treatment and placebo groups, patients with CLE with CLASI-A≥10 showed more response to mezagitamab than placebo.^8^

Lower level of NAD^+^ in CLE skin has additional therapeutic implications. NAD^+^ is a co-factor in multiple cellular oxidation-reduction functions important to cellular homeostasis including the generation of adenoside triphosphate (ATP).^9^ Oral nicotinamide is already being used as a supplement to treat autoimmune skin conditions and prevent non-melanoma skin cancers and can increase circulating levels of NAD^+^.^10^ Nicotinamide prevents ATP depletion, boosts cellular energy, improves protective responses within keratinocytes through enhancing DNA repair and anti-inflammatory effects, and enhances local cutaneous immunity.^11^ A topical version of nicotinamide has shown clinical benefit in patients with discoid lupus.^12^ In addition, NAD^+^ boosting compounds confer anti-inflammatory effects. For example, nicotinamide riboside, an NAD^+^ precursor, can attenuate type I IFN levels in myeloid cells in an NAD^+^-dependent manner, making it a potential adjunct for SLE management.^13^

Cysteine and arabinose were found to be upregulated in CLE sera when compared with control sera. The upregulation of cysteine in CLE sera may reflect buildup of this metabolite from abnormalities in the downstream pathway converting cystine to cysteine and glutathione.^14^ Therapeutic options such as *N*-acetylcysteine have been used to correct this pathway in SLE and reduce oxidative stress, and may have a potential role in treatment of CLE.^15^ Additionally, arabinose is involved in glucose metabolism by selectively inhibiting intestinal sucrose. Since the connection between arabinose and autoimmune disorders has not yet been studied, the significance of the upregulation of arabinose in CLE sera and the correlation with disease activity need to be further investigated.

Limitations include small sample size and unknown specificity of these data to CLE. Since metabolomic analyses were performed in bulk, our findings reflect global skin changes. Future studies will include confirmatory assays including individual cell analyses, with larger and more heterogeneous cohorts to confirm and expand on these observations. Nonetheless, our novel pilot study shows that dysregulated metabolites in CLE skin may be linked to abnormalities in NAD^+^ salvage pathway. This may open up novel therapeutic efforts to correct this pathway, including CD38 blockade and NAD^+^ supplementation (figure 2) for CLE.

## supplementary material

10.1136/lupus-2024-001401 online supplemental file 1
