# Supplementary material for: Metabolic profiles of cutaneous lupus have abnormalities in the nicotinamide adenine dinucleotide pathway
Source: Lupus Sci Med. 2025 Jan 25;12(1):e001401. doi: 10.1136/lupus-2024-001401 (PMC11784198; doi:10.1136/lupus-2024-001401)
Supplement: online supplemental file 1 [file lupus-12-1-s001.docx]

**Supplementary Methods**

*Sample storage and preparation*

Blood samples were collected from patients using SST blood collection tubes and centrifuged at 2500 rpm to separate serum from red blood cells. 50 µL of serum was added to 950 µL of cold 80% methanol. The mixture was vortexed for 1 minute, and then centrifuged at 20,160 g for 15 minutes at 4°C to perform deproteinization. The supernatant was collected in a new tube and frozen at -80°C.

Skin biopsies were collected from patients in cold HEPES Buffered Saline Solution (HBSS) with penicillin, streptomycin, and amphotericin. Skin was rinsed in PBS, and then homogenized with scissors. The homogenate was added to 1 mL of cold 80% methanol. The mixture was vortexed for 1 minute and then centrifuged at 20,160 g for 15 minutes at 4°C. The supernatant was collected in a new tube and frozen at -80°C.

**Supplementary Table 1. Patient Demographic and Clinical Information**

|  | **CLE skin samples (N=5)** | **CLE serum samples (N=9)** | **Control skin samples (N=6)** | **Control serum samples (N=8)** |
| --- | --- | --- | --- | --- |
| *Gender, n (%)* |  |  |  |  |
| Male | 1 (25%) | 2 (22%) | 5 (83%) | 3 (37.5%) |
| Female | 4 (80%) | 7 (78%) | 1 (17%) | 5 (62.5%) |
| *Race/Ethnicity, n (%)* |  |  |  |  |
| White/Hispanic | 1 (20%) | 2 (22%) | 1 (17%) | 2 (25%) |
| Black/Non-Hispanic | 3 (60%) | 6 (67%) | 0 (0%) | 1 (12.5%) |
| White/Non-Hispanic | 1 (20%) | 1 (11%) | 5 (83%) | 1 (12.5%) |
| Asian/Non-Hispanic | 0 (0%) | 0 (0%) | 0 (0%) | 4 (50%) |
| Age, median (IQR) | 39 (28-63) | 54 (39-63) | 62 (53-80.75) | 25 (24-25.25) |
| *Biopsy site, n (%)*  Scalp  Face  Ear  Back  Arm  Leg | 0 (0%)  2 (40%)  0 (0%)  1 (20%)  1 (20%)  1 (20%) | N/A  N/A  N/A  N/A  N/A  N/A | 1 (17%)  3 (50%)  1 (17%)  0 (0%)  0 (0%)  1 (17%) | N/A  N/A  N/A  N/A  N/A  N/A |
| *Predominant CLE subtype, n (%)*  ACLE  CCLE  SCLE | 1 (20%)  4 (80%)  0 (0%) | 1 (11%)  8 (89%)  0 (0%) | N/A  N/A  N/A | N/A  N/A  N/A |
| CLASI-A score, median (IQR) | 7 (4.75-10.25) | 6 (5-10) | N/A | N/A |
| *Current medications, n (%)* |  |  |  |  |
| Topical medications or none | 4 (80%)^a^ | 9 (100%)^b^ | N/A | N/A |
| Immunosuppressive ± anti-malarials ± topical medications | 1 (20%)^c^ | 0 (0%) | N/A | N/A |
| *Laboratory history, n (%)*  Positive anti-dsDNA^d^  Positive anti-SS-A^e^  Positive anti-SS-B^f^  Positive anti-Sm^g^  Positive anti-RNP^h^  Low C3^i^  Low C4^j^ | 1 (33.3%)  1 (33.3%)  0 (0%)  1 (33.3%)  0 (0%)  1 (50%)  1 (50%) | 0 (0%)  1 (33.3%)  1 (33.3%)  1 (25%)  0 (0%)  1 (33.3%)  1 (33.3%) | N/A  N/A  N/A  N/A  N/A  N/A  N/A | N/A  N/A  N/A  N/A  N/A  N/A  N/A |

Abbreviations: ACLE: acute cutaneous lupus erythematosus; CLASI-A: Cutaneous Lupus Erythematosus Disease Area and Severity Index Activity; CLE: cutaneous lupus erythematosus; CCLE: chronic cutaneous lupus erythematosus; IQR: interquartile range; SCLE: subacute cutaneous lupus erythematosus.

^a^ Three patients were on topical corticosteroids, and one patient was not on any medications.

^b^ Three patients were on topical corticosteroids, one patient was on topical immunomodulators, and five patients were not on any medications.

^c^ One patient was on hydroxychloroquine and mycophenolate mofetil.

^d^ Two CLE skin patients and five CLE serum patients did not have anti-dsDNA antibody data.

^e^ Two CLE skin patients and six CLE serum patients did not have anti-SS-A antibody data.

^f^ Two CLE skin patients and six CLE serum patients did not have anti-SS-B antibody data.

^g^ Two CLE skin patients and five CLE serum patients did not have anti-Sm antibody data.

^h^ Two CLE skin patients and five CLE serum patients did not have anti-RNP antibody data.

^i^ Three CLE skin patients and six CLE serum patients did not have C3 data.

^j^ Three CLE skin patients and six CLE serum patients did not have C4 data.

**Supplementary Table 2. Uniquely expressed metabolites in CLE skin samples compared with control skin samples**

| **CLE Mean Peak Intensity** | **Control Mean Peak Intensity** | **Fold change** | **P-Value** | **Adjusted P-Value for Multiple Comparisons** | **Compound Name** | **Formula** | **Mass** |
| --- | --- | --- | --- | --- | --- | --- | --- |
| 8.309602131 | 10.23582359 | 0.811815685 | 2.07498E-05 | 0.006494697* | 4-PYRIDOXATE | C8 H9 N O4 | 183.0529 |
| 14.62324038 | 12.88005274 | 1.135340101 | 0.000110174 | 0.016216477* | N-ACETYLSERINE | C5 H9 N O4 | 147.0533 |
| 20.40804866 | 23.61287589 | 0.864276286 | 0.000187812 | 0.016216477* | NAD+ | C21 H27 N7 O14 P2 | 663.1092 |
| 11.47857548 | 15.54107192 | 0.738596124 | 0.000247127 | 0.016216477* | HIPPURATE | C9 H9 N O3 | 179.0584 |
| 14.66629755 | 19.35629084 | 0.757701859 | 0.000259049 | 0.016216477* | NICOTINAMIDE MONONUCLEOTIDE | C11 H15 N2 O8 P | 334.0574 |
| 17.11986113 | 18.47183523 | 0.926808891 | 0.000337009 | 0.017580649* | PIPECOLATE | C6 H11 N O2 | 129.0797 |
| 21.48789555 | 18.74341502 | 1.146423719 | 0.000617025 | 0.027589821* | CITRULLINE | C6 H13 N3 O3 | 175.0958 |
| 13.4279297 | 11.36823408 | 1.181179909 | 0.000941941 | 0.036853422* | URACIL | C4 H4 N2 O2 | 112.0271 |
| 16.30420643 | 19.69610106 | 0.827788524 | 0.001356303 | 0.04416175* | Cyclic ADP-ribose | C15 H21 N5 O13 P2 | 541.06 |
| 11.29006326 | 14.60041343 | 0.773270107 | 0.001410919 | 0.04416175* | QUINATE | C7 H12 O6 | 192.0635 |
| 13.20945821 | 11.09494453 | 1.190583528 | 0.001720486 | 0.048955651* | THIAMINE PYROPHOSPHATE | C12 H19 N4 O7 P2 S | 425.0488 |
| 12.9133885 | 11.20894062 | 1.15206146 | 0.002442298 | 0.059310877 | DEOXYGUANOSINE | C10 H13 N5 O4 | 267.0965 |
| 13.95058961 | 12.78490211 | 1.091176881 | 0.002584631 | 0.059310877 | SARCOSINE | C3 H7 N O2 | 89.0474 |
| 14.68873246 | 17.73816581 | 0.82808632 | 0.002652883 | 0.059310877 | c | C11 H19 N O6 | 261.1226 |
| 16.32696695 | 13.69723937 | 1.191989605 | 0.002987976 | 0.062349106 | Pyrroline-5-carboxylate | C5 H7 N O2 | 113.0478 |
| 12.05472814 | 13.78303448 | 0.874606253 | 0.004587576 | 0.089744461 | cis-ACONITATE | C6 H6 O6 | 174.0163 |
| 12.69895391 | 15.06058229 | 0.84319143 | 0.00582291 | 0.106156927 | Glycodeoxycholic acid | C26 H43 N O5 | 449.3132 |
| 15.40562092 | 17.72821559 | 0.868988807 | 0.00662495 | 0.106156927 | PANTOTHENATE | C9 H17 N O5 | 219.1106 |
| 22.0693226 | 19.66721739 | 1.122137523 | 0.007403686 | 0.106156927 | OXOPROLINE | C5 H7 N O3 | 129.0432 |
| 10.03127286 | 8.5024088 | 1.179815403 | 0.007595874 | 0.106156927 | 10-HAD | C10 H18 O3 | 186.1252 |
| 14.21242798 | 12.66041952 | 1.122587444 | 0.007645237 | 0.106156927 | ETHANOLAMINE PHOSPHATE | C2 H8 N O4 P | 141.02 |
| 9.021104294 | 10.66214963 | 0.846086821 | 0.00780634 | 0.106156927 | 2-HYDROXY-4-(METHYLTHIO)BUTANOATE | C5 H10 O3 S | 150.0347 |
| 13.85768453 | 10.75043821 | 1.289034387 | 0.008109254 | 0.106156927 | VALINE D8 | C5 H3 D8 N O2 | 125.1298 |
| 13.22988125 | 15.29562528 | 0.864945434 | 0.008139828 | 0.106156927 | 3-METHYL-2-OXOVALERATE | C6 H10 O3 | 130.0634 |
| 12.90240863 | 14.38563782 | 0.896895139 | 0.008565576 | 0.107241013 | 2-KETOHEXANOIC | C6 H10 O3 | 130.0633 |
| 13.97105463 | 12.67224434 | 1.102492522 | 0.008931616 | 0.107522916 | VALINE | C5 H11 N O2 | 117.0797 |
| 22.51482477 | 20.47997416 | 1.099358065 | 0.009818676 | 0.111740924 | O-ACETYLSERINE | C5 H9 N O4 | 147.0534 |
| 16.47604681 | 15.25343635 | 1.080153117 | 0.009995993 | 0.111740924 | PUTRESCINE-NH3 | C4 H9 N | 71.0731 |
| 10.51048686 | 8.68739728 | 1.20985452 | 0.010388416 | 0.112123253 | THYMINE | C5 H6 N2 O2 | 126.0425 |
| 13.29514566 | 16.31444896 | 0.814930722 | 0.011645831 | 0.121504835 | Adipoyl-L-carnitine | C13 H23 N O6 | 289.1527 |
| 17.9708753 | 20.47855877 | 0.877545901 | 0.013065473 | 0.1241507 | GLUCONATE | C6 H12 O7 | 196.0593 |
| 13.91727988 | 15.39844184 | 0.90381092 | 0.01311183 | 0.1241507 | 6-HYDROXYNICOTINATE | C6 H5 N O3 | 139.0269 |
| 16.44391918 | 18.13210906 | 0.906895007 | 0.013262575 | 0.1241507 | GLUTARYLCARNITINE | C12 H21 N O6 | 275.1373 |
| 9.412769912 | 7.933142528 | 1.186512139 | 0.014150512 | 0.1241507 | N-FORMYLGLYCINE | C3 H5 N O3 | 103.028 |
| 13.79633758 | 12.58123689 | 1.096580384 | 0.014350096 | 0.1241507 | HISTIDINE | C6 H9 N3 O2 | 155.07 |
| 18.53502262 | 16.42608466 | 1.128389571 | 0.014492059 | 0.1241507 | THREONINE | C4 H9 N O3 | 119.0581 |
| 13.82922088 | 14.99516167 | 0.922245534 | 0.014675961 | 0.1241507 | trans-2-Octenoyl-L-carnitine | C15 H27 N O4 | 285.1938 |
| 14.86835354 | 13.78715431 | 1.078420768 | 0.015964233 | 0.128239366 | GLUTARATE | C5 H8 O4 | 132.0422 |
| 10.24875238 | 9.287395905 | 1.103511951 | 0.016501751 | 0.128239366 | 3-(2-HYDROXYPHENYL)PROPANOATE | C9 H10 O3 | 166.0626 |
| 15.94863045 | 18.15233067 | 0.878599599 | 0.016576466 | 0.128239366 | N-ACETYLORNITHINE | C7 H14 N2 O3 | 174.1011 |
| 12.22427578 | 13.81965939 | 0.884556951 | 0.016833487 | 0.128239366 | HYDROQUINONE | C6 H6 O2 | 110.0369 |
| 15.64615311 | 14.07205718 | 1.111859688 | 0.017207838 | 0.128239366 | DIMETHYLGLYCINE | C4 H9 N O2 | 103.0632 |
| 19.82582897 | 21.63333205 | 0.916448235 | 0.018436244 | 0.134198707 | CITRATE | C6 H8 O7 | 192.0282 |
| 20.78784914 | 18.7959664 | 1.105973947 | 0.019336786 | 0.13604578 | TRYPTOPHAN D5 | C11 H7 D5 N2 O2 | 209.1222 |
| 17.14174872 | 14.85062728 | 1.154277755 | 0.019799469 | 0.13604578 | GLUTAMATE D3 | C5 H6 D3 N O4 | 150.0726 |
| 14.65091018 | 11.31547262 | 1.294767853 | 0.020865163 | 0.13604578 | N1-ACETYLSPERMINE | C12 H28 N4 O | 244.2264 |
| 12.80160773 | 11.33942562 | 1.128946752 | 0.020926349 | 0.13604578 | MALEIMIDE | C4 H3 N O2 | 97.0167 |
| 11.48425026 | 9.507312437 | 1.207938661 | 0.022158692 | 0.13604578 | METHIONINE SULFOXIDE | C5 H11 N O3 S | 165.0463 |
| 18.69669079 | 17.52882958 | 1.066625167 | 0.022359734 | 0.13604578 | SEBACATE | C10 H18 O4 | 202.121 |
| 10.56800787 | 12.25186608 | 0.862563123 | 0.02279858 | 0.13604578 | 2-ISOPROPYLMALATE | C7 H12 O5 | 176.0684 |
| 19.27335763 | 16.37235336 | 1.177189205 | 0.02288598 | 0.13604578 | S-ADENOSYLMETHIONINE | C15 H22 N6 O5 S | 398.1368 |
| 19.32725372 | 21.55195073 | 0.896775144 | 0.022974588 | 0.13604578 | SALSOLINOL | C10 H13 N O2 | 179.0956 |
| 16.23880599 | 15.15130343 | 1.071776172 | 0.023036506 | 0.13604578 | BENZOATE | C7 H6 O2 | 122.037 |
| 9.88947384 | 12.13548181 | 0.814922225 | 0.024370004 | 0.136395251 | AICAR | C9 H15 N4 O8 P | 338.0602 |
| 20.0594431 | 21.18942844 | 0.946672213 | 0.024394551 | 0.136395251 | O-ACETYLCARNITINE | C9 H17 N O4 | 203.1171 |
| 15.52861426 | 17.4645177 | 0.889152196 | 0.024402984 | 0.136395251 | N-ACETYLASPARTYLGLUTAMATE | C11 H16 N2 O8 | 304.0907 |
| 22.26870315 | 23.83496709 | 0.934287137 | 0.025534777 | 0.140217287 | Butyrylcarnitine | C11 H21 N O4 | 231.1475 |
| 15.38474744 | 12.98005146 | 1.185260897 | 0.0277207 | 0.147386029 | ASPARTATE D3 | C4 H4 D3 N O4 | 136.0567 |
| 14.22359375 | 15.16520147 | 0.937909976 | 0.027782031 | 0.147386029 | 2-HYDROXYGLUTARIC | C5 H8 O5 | 148.037 |
| 19.03342 | 20.55722495 | 0.925874968 | 0.028647967 | 0.147583602 | TRIGONELLINE | C7 H7 N O2 | 137.0478 |
| 11.91487029 | 10.11831497 | 1.177554793 | 0.02915035 | 0.147583602 | CYSTEINE | C3 H7 N O2 S | 121.0201 |
| 20.84443338 | 18.86945904 | 1.104665128 | 0.029582574 | 0.147583602 | KYNURENINE | C10 H12 N2 O3 | 208.0853 |
| 10.30021314 | 9.477151545 | 1.086846938 | 0.029937946 | 0.147583602 | N-ACETYLGLYCINE | C4 H7 N O3 | 117.0432 |
| 12.09859036 | 10.34619136 | 1.16937624 | 0.030176839 | 0.147583602 | THIAMINE MONOPHOSPHATE | C12 H17 N4 O4 P S | 344.0667 |
| 9.312738656 | 8.15835702 | 1.141496827 | 0.031644836 | 0.152382056 | CYSTINE | C6 H12 N2 O4 S2 | 240.0242 |
| 23.28752559 | 22.25372035 | 1.046455389 | 0.032209942 | 0.152753211 | SPERMIDINE | C7 H19 N3 | 145.1581 |
| 13.17893251 | 14.96427394 | 0.88069308 | 0.032911625 | 0.153751322 | 2-HYDROXYBUTYRATE | C4 H8 O3 | 104.0476 |
| 12.51377017 | 10.47096942 | 1.195091845 | 0.03546203 | 0.163229638 | SORBITOL | C6 H14 O6 | 182.0787 |
| 19.19809977 | 18.02627791 | 1.065006313 | 0.037136482 | 0.166220598 | undecanedioic acid | C11 H20 O4 | 216.1368 |
| 14.8975987 | 16.07859441 | 0.926548572 | 0.037173936 | 0.166220598 | PROPIONYLCHOLINE | C8 H17 N O2 | 159.1268 |
| 20.24814196 | 19.57925484 | 1.034163053 | 0.038050992 | 0.167745922 | ARGININE | C6 H14 N4 O2 | 174.1118 |
| 16.85972969 | 15.32635355 | 1.100048334 | 0.039408551 | 0.169429974 | GUANIDINOACETATE | C3 H7 N3 O2 | 117.0547 |
| 15.00477377 | 13.4731991 | 1.11367565 | 0.039515617 | 0.169429974 | 4-HYDROXYBENZALDEHYDE | C7 H6 O2 | 122.0369 |
| 23.15082589 | 22.63163973 | 1.022940722 | 0.042528045 | 0.179882137 | INOSINE | C10 H12 N4 O5 | 268.082 |
| 14.98004915 | 13.46702871 | 1.112349982 | 0.043722377 | 0.182468054 | 4-HYDROXYPHENYLPYRUVATE | C7 H6 O2 | 122.0369 |
| 11.09856031 | 9.892729533 | 1.121890604 | 0.047358707 | 0.195043095 | N-ACETYLLEUCINE | C8 H15 N O3 | 173.1053 |
| 12.05464571 | 14.12415629 | 0.853477225 | 0.049414932 | 0.198824356 | THEOPHYLLINE | C7 H8 N4 O2 | 180.0648 |
| 12.83581927 | 15.01166763 | 0.855056186 | 0.049547284 | 0.198824356 | GLYCOCHOLATE | C26 H43 N O6 | 465.308 |
| 16.10126755 | 13.97343862 | 1.152276686 | 0.051239291 | 0.20110343 | N-ACETYLGLUCOSAMINE | C8 H15 N O6 | 221.0906 |
| 14.04401535 | 13.24364955 | 1.060433931 | 0.051400238 | 0.20110343 | LYSINE | C6 H14 N2 O2 | 146.1053 |
| 24.80850626 | 23.3420324 | 1.062825457 | 0.054440921 | 0.210370474 | BETAINE | C5 H11 N O2 | 117.0793 |
| 18.85516921 | 19.77146498 | 0.953655646 | 0.055567485 | 0.212105156 | LACTATE | C3 H6 O3 | 90.0321 |
| 16.60173535 | 15.57046179 | 1.06623269 | 0.05627919 | 0.212233571 | 3,3-Dimethylglutaric acid | C7 H12 O4 | 160.0737 |
| 14.77367653 | 13.53592295 | 1.091442127 | 0.057832895 | 0.213607139 | OMEGA-HYDROXYDODECANOATE | C12 H24 O3 | 216.1725 |
| 16.57925641 | 15.55422258 | 1.065900679 | 0.058008329 | 0.213607139 | 6-CARBOXYHEXANOATE | C7 H12 O4 | 160.0737 |
| 24.07145136 | 22.08169419 | 1.0901089 | 0.059758204 | 0.217347916 | UROCANATE | C6 H6 N2 O2 | 138.0438 |
| 10.83090907 | 9.673062913 | 1.119697987 | 0.060412999 | 0.217347916 | GLUCONOLACTONE | C6 H10 O6 | 178.0473 |
| 9.298167554 | 10.4190696 | 0.892418221 | 0.061851621 | 0.219994969 | ANSERINE | C10 H16 N4 O3 | 240.1231 |
| 18.5042504 | 17.52177062 | 1.056071946 | 0.063623929 | 0.223756066 | BETAINE ALDEHYDE | C5 H11 N O | 101.084 |
| 17.41106422 | 19.79197451 | 0.879703246 | 0.064552254 | 0.224498395 | THEOBROMINE | C7 H8 N4 O2 | 180.0651 |
| 13.98023065 | 15.10861583 | 0.925315119 | 0.065863759 | 0.226542379 | HYDROXYPHENYLLACTATE | C9 H10 O4 | 182.0579 |
| 21.95904465 | 22.75064873 | 0.965205208 | 0.068807631 | 0.234095528 | CREATININE | C4 H7 N3 O | 113.0591 |
| 20.39596409 | 19.61491204 | 1.039819299 | 0.069892937 | 0.234616663 | GLUTAMATE | C5 H9 N O4 | 147.0542 |
| 12.69269946 | 11.57339356 | 1.096713715 | 0.070459956 | 0.234616663 | 3-Hydroxysebacic acid | C10 H18 O5 | 218.1155 |
| 14.68452509 | 13.85052295 | 1.060214487 | 0.071821142 | 0.236631763 | CORTEXOLONE | C21 H30 O4 | 346.2135 |
| 26.09670645 | 25.57918714 | 1.020232047 | 0.074085093 | 0.237794038 | HYPOXANTHINE | C5 H4 N4 O | 136.0397 |
| 8.517914541 | 9.426048283 | 0.903657003 | 0.074214765 | 0.237794038 | BUTANOATE | C4 H8 O2 | 88.0524 |
| 18.03298291 | 16.8412095 | 1.07076531 | 0.074453086 | 0.237794038 | 5-AMINOLEVULINATE | C5 H9 N O3 | 131.0578 |
| 12.68846716 | 11.29002526 | 1.123865259 | 0.077257793 | 0.244259487 | HYDROXYISOCAPROIC | C6 H12 O3 | 132.0786 |
| 22.19968245 | 20.923927 | 1.060971129 | 0.079464862 | 0.24536459 | PHOSPHORYLCHOLINE | C5 H14 N O4 P | 183.066 |
| 14.71396528 | 13.48840536 | 1.090860253 | 0.080238831 | 0.24536459 | CYTIDINE 2',3'-CYCLIC PHOSPHATE | C9 H12 N3 O7 P | 305.041 |
| 18.53138769 | 17.13753181 | 1.081333526 | 0.080759396 | 0.24536459 | 2-HYDROXYPYRIDINE | C5 H5 N O | 95.0368 |
| 12.6813697 | 11.30013996 | 1.122231206 | 0.081312438 | 0.24536459 | 2-Hydroxyhexanoic acid | C6 H12 O3 | 132.0786 |
| 12.30384638 | 10.36778265 | 1.186738457 | 0.081526893 | 0.24536459 | DL-2-AMINOCTANOATE | C8 H17 N O2 | 159.1257 |
| 15.38430102 | 12.90102967 | 1.192486291 | 0.082451634 | 0.245784394 | BENZYL ALCOHOL | C7 H8 O | 108.0576 |
| 23.33010414 | 24.24378395 | 0.962312822 | 0.084190626 | 0.24697424 | PROPIONYLCARNITINE | C10 H19 N O4 | 217.1319 |
| 18.36220422 | 21.56644133 | 0.851424857 | 0.085063616 | 0.24697424 | URATE | C5 H4 N4 O3 | 168.0293 |
| 12.49765603 | 11.78391801 | 1.060568821 | 0.085217949 | 0.24697424 | N,N,N-TRIMETHYLLYSINE | C9 H20 N2 O2 | 188.1526 |
| 15.27732154 | 13.63643129 | 1.120331355 | 0.087078756 | 0.25005184 | 4-HYDROXY-3-METHOXYPHENYLGLYCOL | C9 H12 O4 | 184.0718 |
| 13.90652179 | 12.46160779 | 1.115949243 | 0.088503897 | 0.251833817 | 4-QUINOLINECARBOXYLATE | C10 H7 N O2 | 173.0478 |
| 10.00977999 | 8.961116131 | 1.1170238 | 0.089421589 | 0.252152769 | ACETOACETATE | C4 H6 O3 | 102.0315 |
| 11.18528891 | 10.16003071 | 1.100910935 | 0.091258706 | 0.255035491 | Phenylacetic acid | C8 H8 O2 | 136.052 |
| 15.07783143 | 13.06632287 | 1.153946033 | 0.094917027 | 0.262911765 | 4-IMIDAZOLEACETATE | C5 H6 N2 O2 | 126.0418 |
| 17.20727826 | 16.11509424 | 1.067773977 | 0.097464087 | 0.267598764 | 5-Methyluridine | C10 H14 N2 O6 | 258.0854 |
| 14.02193048 | 15.96709861 | 0.878176482 | 0.099400165 | 0.267969634 | GLYCOCHENODEOXYCHOLATE | C26 H43 N O5 | 449.314 |
| 17.00519813 | 15.37675541 | 1.105902883 | 0.099400994 | 0.267969634 | 4-GUANIDINOBUTANOATE | C5 H11 N3 O2 | 145.0853 |
| 21.16407438 | 19.33397599 | 1.094657115 | 0.100167563 | 0.267969634 | PYROGLUTAMATE | C5 H7 N O3 | 129.0429 |
| 11.14064336 | 8.962969211 | 1.242963476 | 0.101782549 | 0.269032331 | DEOXYCYTIDINE | C9 H13 N3 O4 | 227.0909 |
| 11.16464389 | 10.39980266 | 1.073543821 | 0.102915736 | 0.269032331 | ACETOIN | C4 H8 O2 | 88.0522 |
| 15.30002302 | 13.67588249 | 1.118759468 | 0.103143386 | 0.269032331 | CYCLIC AMP | C10 H12 N5 O6 P | 329.052 |
| 10.21805836 | 7.702712089 | 1.326553328 | 0.109088338 | 0.28218719 | EPINEPHRINE | C9 H13 N O3 | 183.0894 |
| 11.61436625 | 9.763185156 | 1.189608316 | 0.116752995 | 0.295887091 | METHYL VANILLATE | C9 H10 O4 | 182.0574 |
| 14.10685564 | 15.58038294 | 0.905424192 | 0.117029199 | 0.295887091 | XANTHOSINE | C10 H12 N4 O6 | 284.0755 |
| 13.02456591 | 12.33551018 | 1.055859524 | 0.118376026 | 0.295887091 | SEROTONIN | C10 H12 N2 O | 176.0951 |
| 15.69747764 | 13.98727336 | 1.122268596 | 0.118578379 | 0.295887091 | GLUCOSAMINE | C6 H13 N O5 | 179.0789 |
| 25.88127722 | 24.99929205 | 1.035280406 | 0.119111097 | 0.295887091 | L-CARNITINE | C7 H15 N O3 | 161.1057 |
| 12.5366627 | 10.99173187 | 1.140553904 | 0.121416706 | 0.299222329 | FLAVONE | C15 H10 O2 | 222.0695 |
| 17.2983042 | 19.68671525 | 0.878679047 | 0.122568432 | 0.299222329 | STEAROYLCARNITINE | C25 H49 N O4 | 427.3649 |
| 13.83392082 | 12.89647971 | 1.07268969 | 0.123321663 | 0.299222329 | HOMOSERINE | C4 H9 N O3 | 119.0584 |
| 16.55609394 | 15.83618246 | 1.045459914 | 0.127328681 | 0.306568285 | DEOXYINOSINE | C10 H11 N4 Na O4 | 274.068 |
| 13.05341611 | 12.11591391 | 1.077377754 | 0.134015682 | 0.320205408 | N-ACETYLALANINE | C5 H9 N O3 | 131.0582 |
| 14.00699825 | 15.54418561 | 0.90110853 | 0.13516002 | 0.320493077 | S-ADENOSYLHOMOCYSTEINE | C14 H20 N6 O5 S | 384.1216 |
| 16.95418235 | 19.94426409 | 0.850078111 | 0.136359362 | 0.320905867 | CAFFEINE | C8 H10 N4 O2 | 194.0809 |
| 15.77664391 | 14.58238623 | 1.081897274 | 0.141371775 | 0.330219145 | NICOTINATE | C6 H5 N O2 | 123.0324 |
| 22.46320178 | 21.94839634 | 1.023455264 | 0.150181596 | 0.346594679 | PROLINE | C5 H9 N O2 | 115.0635 |
| 13.26616891 | 11.98862978 | 1.106562564 | 0.150597049 | 0.346594679 | SERINE | C3 H7 N O3 | 105.0429 |
| 15.64701693 | 17.09351929 | 0.915377148 | 0.155302098 | 0.353299624 | GLYCEROL 3-PHOSPHATE | C3 H9 O6 P | 172.0142 |
| 12.2566009 | 11.43191253 | 1.072139142 | 0.155767886 | 0.353299624 | ASPARAGINE | C4 H8 N2 O3 | 132.0537 |
| 14.3870701 | 13.08022097 | 1.099910325 | 0.162428039 | 0.365755225 | 4-ACETYLBUTYRATE | C6 H10 O3 | 130.0628 |
| 17.91044033 | 19.39971789 | 0.923231999 | 0.164029327 | 0.36672271 | TRYPTOPHAN | C11 H12 N2 O2 | 204.0909 |
| 21.22917541 | 21.84256803 | 0.971917559 | 0.168968295 | 0.375085648 | NICOTINAMIDE | C6 H6 N2 O | 122.0488 |
| 16.13748253 | 16.71099312 | 0.96568064 | 0.176855895 | 0.389830247 | BENZYLAMINE | C7 H9 N | 107.0737 |
| 19.41415301 | 21.23270687 | 0.914351294 | 0.1822462 | 0.398902521 | METHIONINE | C5 H11 N O2 S | 149.0514 |
| 13.93704856 | 13.23793061 | 1.052811725 | 0.184857407 | 0.401808113 | 6-HYDROXYDOPAMINE | C8 H11 N O3 | 169.0743 |
| 10.95553897 | 9.184064544 | 1.192885668 | 0.196365167 | 0.423877913 | MANNITOL | C6 H14 O6 | 182.0794 |
| 11.45074731 | 12.7263754 | 0.899765012 | 0.198270498 | 0.425059354 | N-FORMYL-L-METHIONINE | C6 H11 N O3 S | 177.0457 |
| 20.52615228 | 19.42720872 | 1.056567239 | 0.199684059 | 0.425177621 | TAURINE | C2 H7 N O3 S | 125.0157 |
| 10.06020252 | 8.886484028 | 1.132079064 | 0.203157262 | 0.429650155 | HOMOVANILLATE | C9 H10 O4 | 182.0565 |
| 11.25162767 | 12.89593958 | 0.872493826 | 0.209848415 | 0.436337769 | Glycohyodeoxycholic Acid | C26 H43 N O5 | 449.3131 |
| 11.54127177 | 12.22393168 | 0.944153819 | 0.210409088 | 0.436337769 | PANTOLACTONE | C6 H10 O3 | 130.0615 |
| 15.47535423 | 17.61890772 | 0.87833789 | 0.210501607 | 0.436337769 | BETA-NICOTINAMIDE ADENINE DINUCLEOTIDE | C21 H29 N7 O14 P2 | 665.1235 |
| 9.799466951 | 9.221122188 | 1.062719564 | 0.211918462 | 0.436384729 | 2,3-DIHYDROXYBENZOATE | C7 H6 O4 | 154.0265 |
| 11.22532307 | 12.88607681 | 0.871120298 | 0.213572145 | 0.436915565 | Glycoursodeoxycholic acid | C26 H43 N O5 | 449.3132 |
| 10.07585525 | 9.334503704 | 1.079420563 | 0.216035849 | 0.43908585 | 4-ACETAMIDOBUTANOATE | C6 H11 N O3 | 145.0737 |
| 10.43269121 | 9.710754183 | 1.074344074 | 0.218960909 | 0.440959418 | HOMOCYSTEINE | C4 H9 N O2 S | 135.037 |
| 11.87135141 | 12.47849713 | 0.951344643 | 0.219843284 | 0.440959418 | UREA | C H4 N2 O | 60.0321 |
| 9.68776168 | 8.371508809 | 1.157230065 | 0.223656528 | 0.440959418 | FERULATE | C10 H10 O4 | 194.059 |
| 9.819656167 | 9.26948896 | 1.059352485 | 0.223905326 | 0.440959418 | OXOADIPATE | C6 H8 O5 | 160.0379 |
| 15.99250331 | 13.15604113 | 1.215601498 | 0.224001749 | 0.440959418 | 3-HYDROXYBENZYL ALCOHOL | C7 H8 O2 | 124.0525 |
| 16.85985276 | 18.32534257 | 0.920029336 | 0.229418292 | 0.448799533 | LPE(16:0) | C21 H44 N O7 P | 453.2851 |
| 12.9576699 | 11.54494686 | 1.122367219 | 0.243181748 | 0.468835989 | PALMITATE | C16 H32 O2 | 256.2399 |
| 21.47143051 | 19.58907934 | 1.096091865 | 0.244090032 | 0.468835989 | SPERMINE | C10 H26 N4 | 202.2162 |
| 14.35760329 | 13.57988803 | 1.057269637 | 0.244154205 | 0.468835989 | L-ALANINE | C3 H7 N O2 | 89.0481 |
| 16.09101684 | 16.66882654 | 0.96533591 | 0.251474353 | 0.479948003 | N-METHYLGLUTAMATE | C6 H11 N O4 | 161.069 |
| 22.58694335 | 21.87262184 | 1.032658248 | 0.271471602 | 0.513888697 | DEOXYCARNITINE | C7 H15 N O2 | 145.1106 |
| 9.290997283 | 10.15328766 | 0.915072791 | 0.272541609 | 0.513888697 | PYRUVATE | C3 H4 O3 | 88.016 |
| 17.17952544 | 17.52426134 | 0.980328078 | 0.28624561 | 0.528095563 | CYTOSINE | C4 H5 N3 O | 111.0434 |
| 14.11970004 | 13.19024646 | 1.070465217 | 0.287326114 | 0.528095563 | 2-Hydroxyhexadecanoic acid | C16 H32 O3 | 272.2349 |
| 19.63291301 | 19.22651438 | 1.021137405 | 0.288118872 | 0.528095563 | GUANOSINE | C10 H13 N5 O5 | 283.0923 |
| 15.83488129 | 14.60238855 | 1.084403503 | 0.291102725 | 0.528095563 | Fructose 1,6-bisphosphate | C6 H14 O12 P2 | 339.9951 |
| 13.19659821 | 12.57950006 | 1.049055857 | 0.291285544 | 0.528095563 | 10-HYDROXYDECANOATE | C10 H20 O3 | 188.1413 |
| 16.46924231 | 15.37182762 | 1.071391296 | 0.291292034 | 0.528095563 | INOSINE MONOPHOSPHATE | C10 H13 N4 O8 P | 348.0477 |
| 9.165985001 | 8.249943887 | 1.111036042 | 0.292290306 | 0.528095563 | Mesaconic acid | C5 H6 O4 | 130.0267 |
| 14.10206076 | 13.52456693 | 1.042699617 | 0.293573891 | 0.528095563 | N-ACETYLGLUTAMATE | C7 H11 N O5 | 189.0625 |
| 18.92434056 | 18.45254117 | 1.025568261 | 0.29795973 | 0.529998013 | ADENINE | C5 H5 N5 | 135.0552 |
| 13.86890525 | 13.02041887 | 1.065165828 | 0.298435631 | 0.529998013 | 5-METHYLCYTOSINE | C5 H7 N3 O | 125.0589 |
| 7.504013074 | 6.778105287 | 1.107095974 | 0.299711337 | 0.529998013 | Muricholic Acid | C24 H40 O5 | 408.286 |
| 14.15045833 | 14.51902139 | 0.974615159 | 0.301984848 | 0.531018301 | OPHTHALMATE | C11 H19 N3 O6 | 289.1272 |
| 13.14705013 | 12.71849514 | 1.033695416 | 0.305781519 | 0.534690589 | CITRAMALATE | C5 H8 O5 | 148.037 |
| 10.23977435 | 9.567995195 | 1.070211068 | 0.314921833 | 0.547614076 | XANTHURENATE | C10 H7 N O4 | 205.0356 |
| 15.17501369 | 13.80289196 | 1.099408279 | 0.319342073 | 0.551970848 | DIETHANOLAMINE | C4 H11 N O2 | 105.0787 |
| 18.0184681 | 19.09143001 | 0.943798767 | 0.320954295 | 0.551970848 | LPE(18:1) | C23 H46 N O7 P | 479.2996 |
| 10.5734835 | 10.03684644 | 1.0534667 | 0.329357841 | 0.563327893 | RIBOFLAVIN | C17 H20 N4 O6 | 376.1372 |
| 13.25442014 | 14.72343605 | 0.900226014 | 0.331227228 | 0.563404363 | N-ACETYLMETHIONINE | C7 H13 N O3 S | 191.0614 |
| 17.23613596 | 16.40696625 | 1.050537662 | 0.333002579 | 0.563404363 | ASPARTATE | C4 H7 N O4 | 133.038 |
| 9.642614517 | 10.86048762 | 0.887862023 | 0.342951643 | 0.575192546 | 3-METHYL-2-OXINDOLE | C9 H9 N O | 147.0686 |
| 11.40516116 | 12.49843787 | 0.912526932 | 0.343645387 | 0.575192546 | CARNOSINE | C9 H14 N4 O3 | 226.1074 |
| 21.87061814 | 23.14661662 | 0.944873219 | 0.34912837 | 0.580564553 | LPC(16:0) | C24 H50 N O7 P | 495.3316 |
| 17.46348602 | 18.26352264 | 0.956194835 | 0.35079329 | 0.580564553 | GLUTAMINE | C5 H10 N2 O3 | 146.0704 |
| 20.92121332 | 22.14476808 | 0.944747457 | 0.353197582 | 0.580564553 | LPC(18:1) | C26 H52 N O7 P | 521.3464 |
| 18.85631515 | 19.35841685 | 0.974062874 | 0.355218593 | 0.580564553 | octanoylcarnitine | C15 H29 N O4 | 287.21 |
| 13.10886367 | 13.54220614 | 0.9680006 | 0.356687801 | 0.580564553 | FUMARATE | C4 H4 O4 | 116.0107 |
| 19.87702858 | 19.63085405 | 1.012540185 | 0.357983893 | 0.580564553 | 5-AMINOPENTANOATE | C5 H11 N O2 | 117.0796 |
| 13.2360268 | 12.79955505 | 1.034100541 | 0.370700912 | 0.598089616 | PYRIDOXAL | C8 H9 N O3 | 167.0566 |
| 16.9950634 | 16.37817231 | 1.037665441 | 0.374696685 | 0.601436217 | N-ACETYLNEURAMINATE | C11 H19 N O9 | 309.106 |
| 11.74276491 | 12.33659129 | 0.95186463 | 0.37688224 | 0.601857863 | ISOCITRATE | C6 H8 O7 | 192.0268 |
| 9.007446603 | 9.425222653 | 0.955674676 | 0.378886933 | 0.601987868 | 3-METHOXYTYROSINE | C10 H13 N O4 | 211.0842 |
| 17.46212151 | 18.13672431 | 0.962804595 | 0.385422316 | 0.609278712 | FAD | C27 H33 N9 O15 P2 | 785.1535 |
| 14.35515322 | 13.59083879 | 1.056237473 | 0.402248642 | 0.632682537 | STACHYOSE | C24 H42 O21 | 666.219 |
| 23.27375457 | 22.878541 | 1.017274422 | 0.406005836 | 0.635399133 | 8-HYDROXY-DEOXYGUANOSINE | C10 H13 N5 O5 | 283.0921 |
| 24.96454835 | 24.62995396 | 1.013584857 | 0.42582809 | 0.66020253 | CREATINE | C4 H9 N3 O2 | 131.0698 |
| 20.37557177 | 19.83224667 | 1.027396044 | 0.426073198 | 0.66020253 | BIS(2-ETHYLHEXYL)PHTHALATE | C24 H38 O4 | 390.276 |
| 13.51230691 | 12.58757857 | 1.07346356 | 0.430010886 | 0.663021711 | 3-SULFINOALANINE | C3 H7 N O4 S | 153.0097 |
| 13.09362532 | 12.42738009 | 1.053611077 | 0.436112686 | 0.66396398 | 2-Hydroxytetradecanoic acid | C14 H28 O3 | 244.2035 |
| 10.70847146 | 10.23650641 | 1.046106067 | 0.436734605 | 0.66396398 | L-ORNITHINE | C5 H12 N2 O2 | 132.0897 |
| 9.032827235 | 9.810762046 | 0.920705975 | 0.436985878 | 0.66396398 | URSODEOXYCHOLATE | C24 H40 O4 | 392.2912 |
| 14.04205821 | 13.47425742 | 1.042139672 | 0.454690257 | 0.684795376 | HYPOTAURINE | C2 H7 N O2 S | 109.0211 |
| 16.20798504 | 15.85717918 | 1.022122841 | 0.455438131 | 0.684795376 | CYTIDINE MONOPHOSPHATE | C9 H14 N3 O8 P | 323.0519 |
| 13.34544039 | 13.67098316 | 0.976187318 | 0.457381679 | 0.684795376 | ALPHA-KETOISOVALERATE | C5 H8 O3 | 116.0473 |
| 11.53084568 | 11.14447583 | 1.034669181 | 0.460031027 | 0.684795376 | DEOXYADENOSINE MONOPHOSPHATE | C10 H14 N5 O6 P | 331.0678 |
| 14.25207765 | 13.01046145 | 1.095432142 | 0.461635222 | 0.684795376 | 3-METHOXYTYRAMINE | C9 H13 N O2 | 167.0946 |
| 14.23897253 | 15.11231174 | 0.942210085 | 0.476560813 | 0.703601578 | URIDINE 5'-DIPHOSPHATE | C9 H14 N2 O12 P2 | 404.0021 |
| 16.67719372 | 16.31711005 | 1.022067858 | 0.489269102 | 0.718972905 | N-ACETYLASPARTATE | C6 H9 N O5 | 175.0484 |
| 22.75252668 | 22.34557731 | 1.018211629 | 0.497937378 | 0.725607883 | GUANINE | C5 H5 N5 O | 151.0505 |
| 11.87530325 | 13.01724809 | 0.912274482 | 0.49842075 | 0.725607883 | OXOGLUTARATE | C5 H6 O5 | 146.0216 |
| 10.13990221 | 10.54590269 | 0.961501591 | 0.502530346 | 0.728203695 | PYROCATECHOL | C6 H6 O2 | 110.0366 |
| 11.22229211 | 10.83601432 | 1.03564759 | 0.510200302 | 0.731419778 | QUINOLINATE | C7 H5 N O4 | 167.0203 |
| 13.15498246 | 12.61337947 | 1.042938769 | 0.510774993 | 0.731419778 | 3-HYDROXYMETHYLGLUTARATE | C6 H10 O5 | 162.0527 |
| 10.09112867 | 9.698315116 | 1.040503278 | 0.515680888 | 0.731419778 | N-ACETYLPHENYLALANINE | C11 H13 N O3 | 207.0895 |
| 13.98432035 | 14.47800026 | 0.965901375 | 0.517137422 | 0.731419778 | RAFFINOSE | C18 H32 O16 | 504.1675 |
| 17.04622219 | 16.70663568 | 1.020326445 | 0.517663956 | 0.731419778 | TRIMETHYLAMINE | C3 H9 N | 59.0732 |
| 12.15722015 | 12.47947074 | 0.974177544 | 0.525828398 | 0.731419778 | 6-PHOSPHOGLUCONATE | C6 H13 O10 P | 276.0219 |
| 12.68395984 | 14.27857408 | 0.888321184 | 0.526451722 | 0.731419778 | Lactoylglutathione | C13 H21 N3 O8 S | 379.1044 |
| 14.59608251 | 14.2102362 | 1.027152703 | 0.527720938 | 0.731419778 | DIHYDROURACIL | C4 H6 N2 O2 | 114.0435 |
| 23.94391215 | 23.43740025 | 1.021611266 | 0.527976942 | 0.731419778 | GLYCEROPHOSPHOCHOLINE | C8 H20 N O6 P | 257.1032 |
| 18.18836842 | 17.91359964 | 1.015338558 | 0.533982125 | 0.731419778 | D-RIBOSE 5-PHOSPHATE | C5 H11 O8 P | 230.0189 |
| 16.35710946 | 15.97913268 | 1.023654399 | 0.534855981 | 0.731419778 | ADIPATE | C6 H10 O4 | 146.0581 |
| 18.44092229 | 17.96091447 | 1.026725132 | 0.535891753 | 0.731419778 | DEOXYGUANOSINE-MONOPHOSPHATE | C10 H14 N5 O7 P | 347.0642 |
| 22.73215358 | 22.45614594 | 1.012290962 | 0.537735848 | 0.731419778 | ADENOSINE-MONOPHOSPHATE | C10 H14 N5 O7 P | 347.0644 |
| 10.55599063 | 11.09577067 | 0.951352632 | 0.537770122 | 0.731419778 | 3,4-DIHYDROXYBENZOATE | C7 H6 O4 | 154.0267 |
| 25.75346864 | 25.28732862 | 1.018433739 | 0.539801817 | 0.731419778 | ADENOSINE | C10 H13 N5 O4 | 267.0971 |
| 16.41103101 | 16.65760143 | 0.985197723 | 0.543431755 | 0.733164393 | 5-Methylcytidine | C10 H15 N3 O5 | 257.1028 |
| 15.97221568 | 16.25594748 | 0.98254597 | 0.547579379 | 0.735589466 | SUCCINATE | C4 H6 O4 | 118.0266 |
| 18.76615788 | 19.0320109 | 0.98603127 | 0.551869161 | 0.738183964 | MALATE | C4 H6 O5 | 134.0227 |
| 8.133615833 | 7.627004047 | 1.066423432 | 0.555256256 | 0.739489497 | (3ALPHA,5BETA,7ALPHA)-3,7-DIHYDROXY-12-OXOCHOLAN-24-OIC ACID | C24 H38 O5 | 406.2703 |
| 20.67216023 | 21.32609641 | 0.96933634 | 0.557570356 | 0.739489497 | URIDINE DIPHOSPHATE-N-ACETYLGLUCOSAMINE | C17 H27 N3 O17 P2 | 607.0805 |
| 14.77445925 | 14.25403448 | 1.0365107 | 0.573430137 | 0.755179306 | NORMETANEPHRINE | C9 H13 N O3 | 183.0902 |
| 9.39546451 | 8.819090271 | 1.065355294 | 0.579357986 | 0.755179306 | Ursodeoxycholic acid | C24 H40 O4 | 392.2913 |
| 13.78512502 | 14.164701 | 0.973202683 | 0.58202455 | 0.755179306 | AMINOADIPATE | C6 H11 N O4 | 161.069 |
| 11.05064499 | 10.46883816 | 1.05557511 | 0.58225342 | 0.755179306 | HEXOSE | C6 H12 O6 | 180.0641 |
| 16.35413302 | 16.9720143 | 0.96359411 | 0.582471319 | 0.755179306 | 3-PHOSPHOGLYCERATE | C3 H7 O7 P | 185.9936 |
| 13.34184274 | 14.26693552 | 0.93515827 | 0.583876652 | 0.755179306 | 2-QUINOLINECARBOXYLATE | C10 H7 N O2 | 173.0479 |
| 13.46477931 | 13.18247735 | 1.02141494 | 0.586599764 | 0.75557912 | Traumatic acid | C12 H20 O4 | 228.1358 |
| 20.084321 | 20.47305831 | 0.98101225 | 0.59658731 | 0.764685726 | hexanoylcarnitine | C13 H25 N O4 | 259.1789 |
| 19.68090684 | 20.15238739 | 0.976604233 | 0.60070898 | 0.764685726 | 4-HYDROXYBENZOATE | C7 H6 O3 | 138.0319 |
| 19.69351602 | 20.15667514 | 0.977022048 | 0.60459224 | 0.764685726 | SALICYLATE | C7 H6 O3 | 138.0319 |
| 12.29890068 | 13.17388497 | 0.933581909 | 0.606018572 | 0.764685726 | TRANS-ACONITATE | C6 H6 O6 | 174.0161 |
| 12.69829719 | 13.08287535 | 0.970604462 | 0.607656474 | 0.764685726 | 3-Hydroxyoctanoic acid | C8 H16 O3 | 160.1098 |
| 15.65971955 | 15.16617197 | 1.03254266 | 0.608470886 | 0.764685726 | GUANOSINE MONOPHOSPHATE | C10 H14 N5 O8 P | 363.0575 |
| 10.3433302 | 10.53978869 | 0.981360301 | 0.612169512 | 0.764685726 | MEVALONATE | C6 H12 O4 | 148.0739 |
| 13.16588096 | 13.51538213 | 0.97414049 | 0.613214432 | 0.764685726 | GUANIDINOSUCCINATE | C5 H9 N3 O4 | 175.0593 |
| 10.15766677 | 9.409908667 | 1.079464969 | 0.619253735 | 0.769152456 | Tauro-?-muricholic acid | C26 H45 N O7 S | 515.2881 |
| 13.37653609 | 13.65517706 | 0.979594481 | 0.623470144 | 0.771328676 | GLYCERATE | C3 H6 O4 | 106.0266 |
| 12.30766646 | 12.5862918 | 0.977862793 | 0.635332953 | 0.782910293 | GLUCURONATE | C6 H10 O7 | 194.0432 |
| 15.73096047 | 15.51113976 | 1.014171796 | 0.648145654 | 0.795567018 | FRUCTOSE 6-PHOSPHATE | C6 H13 O9 P | 260.0299 |
| 15.20107938 | 15.43847152 | 0.984623339 | 0.652202657 | 0.797419655 | 1-METHYL-L-HISTIDINE | C7 H11 N3 O2 | 169.0851 |
| 8.008482847 | 8.48434744 | 0.943912647 | 0.659217561 | 0.802860298 | EICOSAPENTAENOATE | C20 H30 O2 | 302.2236 |
| 12.8695664 | 12.63490246 | 1.018572675 | 0.666916686 | 0.809088847 | 7-METHYLGUANOSINE | C11 H15 N5 O5 | 297.107 |
| 12.16022098 | 11.9540067 | 1.017250641 | 0.669562196 | 0.809162036 | 10-Undecenoic acid | C11 H20 O2 | 184.1464 |
| 15.81859228 | 16.17809377 | 0.977778501 | 0.675614981 | 0.813336496 | 1-METHYLADENOSINE | C11 H15 N5 O4 | 281.1132 |
| 22.99183678 | 23.40116751 | 0.982508106 | 0.691899699 | 0.828186091 | Isovalerylcarnitine | C12 H23 N O4 | 245.163 |
| 18.169845 | 17.91473889 | 1.014240013 | 0.693242032 | 0.828186091 | SUBERATE | C8 H14 O4 | 174.0895 |
| 8.726140047 | 9.040363035 | 0.965242216 | 0.725908523 | 0.861851192 | Murideoxycholic Acid | C24 H40 O4 | 392.2916 |
| 14.95644631 | 15.33158189 | 0.975531841 | 0.727051466 | 0.861851192 | DEOXYCHOLATE | C24 H40 O4 | 392.2917 |
| 14.95896005 | 15.33051995 | 0.975763386 | 0.729682319 | 0.861851192 | Deoxycholic acid | C24 H40 O4 | 392.2917 |
| 23.18674335 | 23.76682432 | 0.975592828 | 0.734928381 | 0.864784147 | 2',4'-DIHYDROXYACETOPHENONE | C8 H8 O3 | 152.0476 |
| 15.50110735 | 15.70869199 | 0.986785364 | 0.742764247 | 0.870731121 | 3-METHYLHISTAMINE | C6 H11 N3 | 125.095 |
| 18.45861687 | 18.63674585 | 0.990442056 | 0.746088331 | 0.871364357 | TYROSINE | C9 H11 N O3 | 181.0746 |
| 22.71210664 | 22.60656364 | 1.004668688 | 0.761455018 | 0.877800711 | LEUCINE | C6 H13 N O2 | 131.0953 |
| 18.03217482 | 18.30198289 | 0.985257987 | 0.762043421 | 0.877800711 | URIDINE DIPHOSPHATE GLUCOSE | C15 H24 N2 O17 P2 | 566.054 |
| 16.51137223 | 16.80772918 | 0.982367817 | 0.766401477 | 0.877800711 | XANTHINE | C5 H4 N4 O2 | 152.0335 |
| 16.90949811 | 16.71891401 | 1.011399311 | 0.767088874 | 0.877800711 | DEOXYADENOSINE | C10 H13 N5 O3 | 251.1032 |
| 24.26312864 | 24.32676446 | 0.997384123 | 0.770453249 | 0.877800711 | PHENYLALANINE | C9 H11 N O2 | 165.08 |
| 10.67796173 | 10.35809379 | 1.030880966 | 0.772259062 | 0.877800711 | DOCOSAHEXAENOATE | C22 H32 O2 | 328.2389 |
| 14.38337377 | 14.21207874 | 1.012052778 | 0.772815871 | 0.877800711 | N-ALPHA-ACETYLLYSINE | C8 H16 N2 O3 | 188.1163 |
| 20.85392871 | 21.15700775 | 0.985674768 | 0.774354393 | 0.877800711 | N,N-DIMETHYLARGININE | C8 H18 N4 O2 | 202.1432 |
| 10.79701009 | 10.93066113 | 0.987772832 | 0.776839607 | 0.877800711 | N-ACETYLASPARAGINE | C6 H10 N2 O4 | 174.0653 |
| 11.42717613 | 11.54542128 | 0.989758264 | 0.786965248 | 0.884481948 | CITICOLINE | C14 H26 N4 O11 P2 | 488.1056 |
| 21.82182257 | 21.61663041 | 1.009492329 | 0.790161414 | 0.884481948 | HISTAMINE | C5 H9 N3 | 111.0796 |
| 18.95005904 | 18.80326872 | 1.007806639 | 0.791229858 | 0.884481948 | URIDINE | C9 H12 N2 O6 | 244.0699 |
| 10.94308215 | 11.23696207 | 0.973847031 | 0.79585384 | 0.886484882 | LACTOSE | C12 H22 O11 | 342.1158 |
| 9.611814944 | 9.820824391 | 0.978717729 | 0.816028003 | 0.904629873 | PHENYLLACTATE | C9 H10 O3 | 166.0625 |
| 10.23181231 | 10.08885527 | 1.014169798 | 0.819456969 | 0.904629873 | 2,5-DIHYDROXYBENZOATE | C7 H6 O4 | 154.0266 |
| 6.48003432 | 6.702586563 | 0.966796066 | 0.820975233 | 0.904629873 | GAMMA-LINOLENATE | C18 H30 O2 | 278.224 |
| 10.07837106 | 9.972334332 | 1.01063309 | 0.825609319 | 0.904629873 | 5-Aminopentanoic acid | C5 H11 N O2 | 117.0791 |
| 7.548739686 | 7.403551199 | 1.019610655 | 0.826594708 | 0.904629873 | THYROXINE | C15 H11 I4 N O4 | 776.6822 |
| 10.95095093 | 10.75799598 | 1.017935957 | 0.833693957 | 0.909220239 | 7-Hydroxy-3-oxo-Cholest-4-en-26-oic Acid | C27 H42 O4 | 430.3069 |
| 10.11153626 | 10.39560071 | 0.972674551 | 0.839608395 | 0.912491068 | LINOLEATE | C18 H32 O2 | 280.2399 |
| 18.97125164 | 18.66198114 | 1.016572222 | 0.850182141 | 0.918225468 | METHYLTHIOADENOSINE | C11 H15 N5 O3 S | 297.0902 |
| 13.21883275 | 13.14238049 | 1.005817231 | 0.850752031 | 0.918225468 | D-GLUCURONOLACTONE | C6 H8 O6 | 176.0276 |
| 21.72976888 | 21.66303132 | 1.003080712 | 0.861803003 | 0.926956494 | ISOLEUCINE | C6 H13 N O2 | 131.0956 |
| 18.69850417 | 19.15034516 | 0.976405595 | 0.867711958 | 0.928971779 | GLUTATHIONE REDUCED | C10 H17 N3 O6 S | 307.0844 |
| 11.72209484 | 11.63789093 | 1.007235324 | 0.86961256 | 0.928971779 | GLYCERALDEHYDE 3-PHOSPHATE | C3 H7 O6 P | 169.9973 |
| 11.37854951 | 11.17298631 | 1.018398232 | 0.881268724 | 0.938221465 | CHOLATE | C24 H40 O5 | 408.2862 |
| 10.15724537 | 10.29210271 | 0.986897007 | 0.891791056 | 0.944803984 | Chenodeoxycholic acid | C24 H40 O4 | 392.2909 |
| 14.69089059 | 14.51294471 | 1.012261184 | 0.893488752 | 0.944803984 | TRANS-CINNAMALDEHYDE | C9 H8 O | 132.0576 |
| 10.98528289 | 11.0496232 | 0.994177149 | 0.898836748 | 0.94725893 | 2-Ethyl-2-hydroxybutyric acid | C6 H12 O3 | 132.0786 |
| 9.492729445 | 9.404900851 | 1.009338599 | 0.912957731 | 0.956744788 | GLYCOLATE | C2 H4 O3 | 76.0164 |
| 18.41522945 | 18.31852871 | 1.005278848 | 0.913951092 | 0.956744788 | CDP-ETHANOLAMINE | C11 H20 N4 O11 P2 | 446.0607 |
| 19.69199762 | 19.62082694 | 1.003627303 | 0.919478598 | 0.959322671 | AZELATE | C9 H16 O4 | 188.1058 |
| 22.70723687 | 22.77093962 | 0.997202454 | 0.925928222 | 0.962842304 | OXIDIZED GLUTATHIONE | C20 H32 N6 O12 S2 | 612.1522 |
| 9.174917235 | 9.230072217 | 0.994024426 | 0.93827596 | 0.972451574 | Glycolithocholic Acid | C26 H43 N O4 | 433.3179 |
| 10.90352217 | 10.84263051 | 1.005615948 | 0.949492849 | 0.979841605 | MANDELATE | C8 H8 O3 | 152.0474 |
| 14.57096645 | 14.62779732 | 0.996114871 | 0.952397017 | 0.979841605 | N-ACETYLPUTRESCINE | C6 H14 N2 O | 130.1107 |
| 17.60227631 | 17.63779829 | 0.997986031 | 0.957601547 | 0.979841605 | 3-METHYLADENINE | C6 H7 N5 | 149.0709 |
| 10.25799201 | 10.21355992 | 1.004350304 | 0.961884831 | 0.979841605 | ALLANTOIN | C4 H6 N4 O3 | 158.0439 |
| 14.3951599 | 14.35327872 | 1.002917883 | 0.963622028 | 0.979841605 | HYDROXYKYNURENINE | C10 H12 N2 O4 | 224.08 |
| 12.81965929 | 12.78318998 | 1.002852912 | 0.964189183 | 0.979841605 | SEDOHEPTULOSE-7-PHOSPHATE | C7 H15 O10 P | 290.041 |
| 16.69657051 | 16.74024679 | 0.997390942 | 0.968263161 | 0.980797312 | URIDINE MONOPHOSPHATE | C9 H13 N2 O9 P | 324.0361 |
| 11.69759103 | 11.65822299 | 1.003376848 | 0.974192882 | 0.983620555 | OLEATE | C18 H34 O2 | 282.2556 |
| 8.547633969 | 8.559272637 | 0.998640227 | 0.984955447 | 0.988318834 | ARABINOSE | C5 H10 O5 | 150.053 |
| 13.07959062 | 13.09064879 | 0.999155262 | 0.985161266 | 0.988318834 | 7-HYDROXYOCTANOATE | C8 H16 O3 | 160.1099 |
| 14.69410255 | 14.69553845 | 0.99990229 | 0.999584246 | 0.999584246 | ASCORBATE | C6 H8 O6 | 176.0327 |

**Supplementary Table 3. Uniquely expressed metabolites in CLE serum samples compared with control serum samples**

| **CLE Mean Peak Intensity** | **Control Mean Peak Intensity** | **Fold Change** | **P-Value** | **Adjusted P-Value for Multiple Comparisons** | **Compound Name** | **Formula** | **Mass** |
| --- | --- | --- | --- | --- | --- | --- | --- |
| 12.82095574 | 10.98716384 | 1.166903118 | 6.6724E-05 | 0.0208846* | ARABINOSE | C5 H10 O5 | 150.053 |
| 13.65273044 | 13.08430416 | 1.043443371 | 0.000186471 | 0.029182773* | CYSTINE | C6 H12 N2 O4 S2 | 240.0242 |
| 10.40984605 | 9.69790065 | 1.073412321 | 0.001094995 | 0.113819169 | RIBOFLAVIN | C17 H20 N4 O6 | 376.1372 |
| 10.59170472 | 11.79709689 | 0.897822982 | 0.001712839 | 0.113819169 | 2,3-DIHYDROXYBENZOATE | C7 H6 O4 | 154.0265 |
| 14.92271762 | 14.30080957 | 1.043487611 | 0.001818198 | 0.113819169 | GLUCURONATE | C6 H10 O7 | 194.0432 |
| 16.42161886 | 14.96802114 | 1.097113553 | 0.00222 | 0.115809985 | SPERMIDINE | C7 H19 N3 | 145.1581 |
| 12.30545636 | 10.7088644 | 1.149090687 | 0.003226463 | 0.144268976 | URIDINE DIPHOSPHATE-N-ACETYLGLUCOSAMINE | C17 H27 N3 O17 P2 | 607.0805 |
| 18.37736003 | 17.67624912 | 1.039664009 | 0.00390028 | 0.152598456 | GLUCONATE | C6 H12 O7 | 196.0593 |
| 15.50883866 | 14.7340036 | 1.052588223 | 0.005539095 | 0.192637419 | N-ACETYLNEURAMINATE | C11 H19 N O9 | 309.106 |
| 19.70059432 | 19.16133177 | 1.02814327 | 0.0069253 | 0.216527502 | 5-AMINOLEVULINATE | C5 H9 N O3 | 131.0578 |
| 16.05924727 | 14.64508545 | 1.09656221 | 0.007630631 | 0.216527502 | O-Succinyl-L-carnitine | C11 H19 N O6 | 261.1226 |
| 11.42503946 | 10.86014577 | 1.052015296 | 0.008577982 | 0.216527502 | 6-PHOSPHOGLUCONATE | C6 H13 O10 P | 276.0219 |
| 12.03568843 | 11.33583439 | 1.061738203 | 0.00906426 | 0.216527502 | RAFFINOSE | C18 H32 O16 | 504.1675 |
| 17.979804 | 17.36395393 | 1.035467157 | 0.009684936 | 0.216527502 | MALATE | C4 H6 O5 | 134.0227 |
| 12.14666894 | 11.52019756 | 1.054380264 | 0.010546393 | 0.220068059 | N-ACETYLGLUCOSAMINE | C8 H15 N O6 | 221.0906 |
| 11.41610726 | 12.32281183 | 0.926420643 | 0.013188043 | 0.255405828 | Mesaconic acid | C5 H6 O4 | 130.0267 |
| 17.34738795 | 16.4081532 | 1.057241954 | 0.014807526 | 0.255405828 | NICOTINAMIDE | C6 H6 N2 O | 122.0488 |
| 14.10913475 | 13.57481764 | 1.039360905 | 0.014936557 | 0.255405828 | ISOCITRATE | C6 H8 O7 | 192.0268 |
| 9.527865828 | 8.81375748 | 1.08102201 | 0.016242445 | 0.255405828 | URIDINE DIPHOSPHATE GLUCOSE | C15 H24 N2 O17 P2 | 566.054 |
| 20.91173049 | 20.04208903 | 1.043390759 | 0.016376864 | 0.255405828 | KYNURENINE | C10 H12 N2 O3 | 208.0853 |
| 16.31011012 | 15.73212711 | 1.036739025 | 0.017135854 | 0.255405828 | DIHYDROURACIL | C4 H6 N2 O2 | 114.0435 |
| 15.44615951 | 14.77040404 | 1.045750642 | 0.018183392 | 0.25726644 | GLYCERATE | C3 H6 O4 | 106.0266 |
| 25.37085685 | 24.45094362 | 1.037622811 | 0.0195735 | 0.25726644 | HYPOXANTHINE | C5 H4 N4 O | 136.0397 |
| 15.72775159 | 16.86326323 | 0.932663588 | 0.020087715 | 0.25726644 | HYDROQUINONE | C6 H6 O2 | 110.0369 |
| 23.1488809 | 22.60384041 | 1.024112739 | 0.020548438 | 0.25726644 | ISOLEUCINE | C6 H13 N O2 | 131.0956 |
| 14.00750241 | 13.29962996 | 1.053224973 | 0.021603058 | 0.260067582 | CYSTEINE | C3 H7 N O2 S | 121.0201 |
| 14.91132392 | 14.05625562 | 1.060831869 | 0.023472555 | 0.26673286 | 5-Methyluridine | C10 H14 N2 O6 | 258.0854 |
| 17.65269536 | 16.74959804 | 1.053917552 | 0.023861086 | 0.26673286 | DIMETHYLGLYCINE | C4 H9 N O2 | 103.0632 |
| 19.20774034 | 18.57403452 | 1.034117834 | 0.030713123 | 0.331489911 | GLUTARYLCARNITINE | C12 H21 N O6 | 275.1373 |
| 19.05227998 | 18.21254575 | 1.04610746 | 0.035407634 | 0.367269191 | GLUTAMATE | C5 H9 N O4 | 147.0542 |
| 14.71258145 | 14.29173091 | 1.029447136 | 0.040455196 | 0.367269191 | N-ACETYLALANINE | C5 H9 N O3 | 131.0582 |
| 16.21922749 | 15.79185065 | 1.027063126 | 0.042023113 | 0.367269191 | SUCCINATE | C4 H6 O4 | 118.0266 |
| 13.39593805 | 12.13838953 | 1.103600937 | 0.044011818 | 0.367269191 | FRUCTOSE 6-PHOSPHATE | C6 H13 O9 P | 260.0299 |
| 11.24074529 | 10.69677806 | 1.050853371 | 0.045654983 | 0.367269191 | URIDINE 5'-DIPHOSPHATE | C9 H14 N2 O12 P2 | 404.0021 |
| 20.78917083 | 24.25816188 | 0.856996953 | 0.045656087 | 0.367269191 | CAFFEINE | C8 H10 N4 O2 | 194.0809 |
| 17.88912457 | 18.94979399 | 0.944027391 | 0.04682225 | 0.367269191 | 3-Hydroxysebacic acid | C10 H18 O5 | 218.1155 |
| 16.92620493 | 18.61137696 | 0.909454736 | 0.048128555 | 0.367269191 | HIPPURATE | C9 H9 N O3 | 179.0584 |
| 13.60578386 | 13.93765769 | 0.976188695 | 0.050347947 | 0.367269191 | ASPARAGINE | C4 H8 N2 O3 | 132.0537 |
| 11.09904055 | 12.29294954 | 0.902878558 | 0.051482519 | 0.367269191 | OXIDIZED GLUTATHIONE | C20 H32 N6 O12 S2 | 612.1522 |
| 15.42994032 | 14.97492834 | 1.030384919 | 0.055548231 | 0.367269191 | TRANS-CINNAMALDEHYDE | C9 H8 O | 132.0576 |
| 13.76696989 | 13.15997515 | 1.046124307 | 0.056143487 | 0.367269191 | 4-HYDROXYBENZALDEHYDE | C7 H6 O2 | 122.0369 |
| 13.74613301 | 13.1373972 | 1.046336104 | 0.056197079 | 0.367269191 | 4-HYDROXYPHENYLPYRUVATE | C7 H6 O2 | 122.0369 |
| 11.3121683 | 11.62878476 | 0.972773039 | 0.056728783 | 0.367269191 | METHIONINE SULFOXIDE | C5 H11 N O3 S | 165.0463 |
| 13.39305468 | 15.74481146 | 0.850632903 | 0.058001967 | 0.367269191 | OXOGLUTARATE | C5 H6 O5 | 146.0216 |
| 15.9005131 | 15.14841025 | 1.049648962 | 0.060027438 | 0.367269191 | N-ALPHA-ACETYLLYSINE | C8 H16 N2 O3 | 188.1163 |
| 15.77522118 | 15.17668427 | 1.039437924 | 0.060205557 | 0.367269191 | 7-Hydroxy-3-oxo-Cholest-4-en-26-oic Acid | C27 H42 O4 | 430.3069 |
| 21.57687751 | 21.08457972 | 1.023348713 | 0.060533641 | 0.367269191 | ARGININE | C6 H14 N4 O2 | 174.1118 |
| 14.96024754 | 17.66393886 | 0.846937235 | 0.060592368 | 0.367269191 | THEOPHYLLINE | C7 H8 N4 O2 | 180.0648 |
| 10.48918651 | 9.241334661 | 1.135029397 | 0.063028017 | 0.367269191 | SEDOHEPTULOSE-7-PHOSPHATE | C7 H15 O10 P | 290.041 |
| 14.36445037 | 17.13691788 | 0.838216678 | 0.063117265 | 0.367269191 | QUINATE | C7 H12 O6 | 192.0635 |
| 21.9596417 | 21.43042444 | 1.02469467 | 0.064144648 | 0.367269191 | Isovalerylcarnitine | C12 H23 N O4 | 245.163 |
| 12.64733398 | 12.03697376 | 1.050707115 | 0.066814703 | 0.367269191 | SALICYLATE | C7 H6 O3 | 138.0319 |
| 11.6025034 | 11.20706116 | 1.035285097 | 0.066870155 | 0.367269191 | N-ACETYLPHENYLALANINE | C11 H13 N O3 | 207.0895 |
| 12.99631488 | 12.69165323 | 1.024004883 | 0.066915961 | 0.367269191 | N-ACETYLGLUTAMATE | C7 H11 N O5 | 189.0625 |
| 19.43071103 | 22.30919261 | 0.870973296 | 0.067198415 | 0.367269191 | THEOBROMINE | C7 H8 N4 O2 | 180.0651 |
| 12.43663746 | 11.76757671 | 1.05685629 | 0.069349134 | 0.367269191 | 4-HYDROXYBENZOATE | C7 H6 O3 | 138.0319 |
| 22.40363375 | 22.03331653 | 1.016807148 | 0.07008235 | 0.367269191 | CITRATE | C6 H8 O7 | 192.0282 |
| 12.78821177 | 12.04669835 | 1.061553248 | 0.070383396 | 0.367269191 | XANTHOSINE | C10 H12 N4 O6 | 284.0755 |
| 17.04469039 | 16.35879509 | 1.041928229 | 0.070691601 | 0.367269191 | METHYLTHIOADENOSINE | C11 H15 N5 O3 S | 297.0902 |
| 11.87504215 | 13.13918133 | 0.903788589 | 0.071269477 | 0.367269191 | Glycolithocholic Acid | C26 H43 N O4 | 433.3179 |
| 14.62957293 | 14.19064598 | 1.030930724 | 0.071576424 | 0.367269191 | 2',4'-DIHYDROXYACETOPHENONE | C8 H8 O3 | 152.0476 |
| 11.68681123 | 11.05184944 | 1.05745299 | 0.076707724 | 0.387250282 | S-ADENOSYLHOMOCYSTEINE | C14 H20 N6 O5 S | 384.1216 |
| 19.25628215 | 18.80972223 | 1.02374091 | 0.079312595 | 0.388842937 | PYROGLUTAMATE | C5 H7 N O3 | 129.0429 |
| 13.02309477 | 12.60003991 | 1.033575677 | 0.079507821 | 0.388842937 | cis-ACONITATE | C6 H6 O6 | 174.0163 |
| 10.94791814 | 10.09632964 | 1.084346345 | 0.082408197 | 0.393548444 | 10-HAD | C10 H18 O3 | 186.1252 |
| 23.00726584 | 22.53829515 | 1.020807727 | 0.083904517 | 0.393548444 | PROPIONYLCARNITINE | C10 H19 N O4 | 217.1319 |
| 15.64715178 | 15.3084256 | 1.022126781 | 0.084241999 | 0.393548444 | Pyrroline-5-carboxylate | C5 H7 N O2 | 113.0478 |
| 24.35602875 | 23.98432196 | 1.015497907 | 0.088542925 | 0.407557874 | LEUCINE | C6 H13 N O2 | 131.0953 |
| 23.70431468 | 23.31618474 | 1.016646374 | 0.091283595 | 0.409481416 | PROLINE | C5 H9 N O2 | 115.0635 |
| 10.29831504 | 9.94938921 | 1.035070076 | 0.09274913 | 0.409481416 | Cyclic ADP-ribose | C15 H21 N5 O13 P2 | 541.06 |
| 19.37985524 | 18.99774393 | 1.02011351 | 0.093535529 | 0.409481416 | undecanedioic acid | C11 H20 O4 | 216.1368 |
| 18.89214789 | 18.46343468 | 1.02321958 | 0.094193808 | 0.409481416 | SEBACATE | C10 H18 O4 | 202.121 |
| 20.95974514 | 20.33219437 | 1.030864881 | 0.096933698 | 0.41561983 | O-ACETYLSERINE | C5 H9 N O4 | 147.0534 |
| 21.51105926 | 21.27866896 | 1.01092128 | 0.100198628 | 0.423813115 | LACTATE | C3 H6 O3 | 90.0321 |
| 25.61758664 | 25.30570201 | 1.012324678 | 0.106354644 | 0.438492866 | PHENYLALANINE | C9 H11 N O2 | 165.08 |
| 22.17889418 | 21.70480964 | 1.021842372 | 0.107969332 | 0.438492866 | METHIONINE | C5 H11 N O2 S | 149.0514 |
| 15.9301771 | 15.05482925 | 1.05814399 | 0.108147511 | 0.438492866 | D-GLUCURONOLACTONE | C6 H8 O6 | 176.0276 |
| 15.99056847 | 15.81178005 | 1.011307292 | 0.109599513 | 0.438492866 | 3,3-Dimethylglutaric acid | C7 H12 O4 | 160.0737 |
| 20.7095055 | 20.19431861 | 1.025511477 | 0.110673918 | 0.438492866 | BIS(2-ETHYLHEXYL)PHTHALATE | C24 H38 O4 | 390.276 |
| 12.20033655 | 11.95617131 | 1.020421692 | 0.113985766 | 0.439740367 | 4-ACETYLBUTYRATE | C6 H10 O3 | 130.0628 |
| 13.32911375 | 12.24745423 | 1.08831709 | 0.117037043 | 0.439740367 | D-RIBOSE 5-PHOSPHATE | C5 H11 O8 P | 230.0189 |
| 12.50727917 | 11.26797858 | 1.109984287 | 0.117050781 | 0.439740367 | FLAVONE | C15 H10 O2 | 222.0695 |
| 16.71267024 | 16.03066673 | 1.042543677 | 0.117490211 | 0.439740367 | PROPIONYLCHOLINE | C8 H17 N O2 | 159.1268 |
| 21.24793506 | 22.67154897 | 0.93720703 | 0.118623643 | 0.439740367 | TRIGONELLINE | C7 H7 N O2 | 137.0478 |
| 12.16997164 | 11.34125766 | 1.07307073 | 0.120715731 | 0.439740367 | 3-PHOSPHOGLYCERATE | C3 H7 O7 P | 185.9936 |
| 16.34155406 | 15.94509973 | 1.02486371 | 0.120823232 | 0.439740367 | 5-Methylcytidine | C10 H15 N3 O5 | 257.1028 |
| 11.46391135 | 11.15948226 | 1.027279857 | 0.122693373 | 0.441414088 | N-ACETYLLEUCINE | C8 H15 N O3 | 173.1053 |
| 10.43547012 | 11.25967831 | 0.926800023 | 0.126022798 | 0.448240178 | 2-ISOPROPYLMALATE | C7 H12 O5 | 176.0684 |
| 22.41608035 | 21.78466888 | 1.028984212 | 0.130677137 | 0.45227716 | Butyrylcarnitine | C11 H21 N O4 | 231.1475 |
| 18.13095077 | 17.67217112 | 1.02596057 | 0.131322212 | 0.45227716 | 3-METHYLADENINE | C6 H7 N5 | 149.0709 |
| 15.97581594 | 15.81038334 | 1.010463541 | 0.134834153 | 0.45227716 | 6-CARBOXYHEXANOATE | C7 H12 O4 | 160.0737 |
| 16.28522719 | 15.8666426 | 1.026381422 | 0.136212213 | 0.45227716 | 5-METHYLCYTOSINE | C5 H7 N3 O | 125.0589 |
| 12.30241093 | 12.74551405 | 0.965234582 | 0.136352451 | 0.45227716 | MEVALONATE | C6 H12 O4 | 148.0739 |
| 15.25489367 | 14.96056188 | 1.019673846 | 0.139037445 | 0.45227716 | 2-HYDROXYGLUTARIC | C5 H8 O5 | 148.037 |
| 16.13181878 | 15.50727033 | 1.040274557 | 0.141044264 | 0.45227716 | XANTHINE | C5 H4 N4 O2 | 152.0335 |
| 9.634442732 | 10.18219797 | 0.946204617 | 0.141164334 | 0.45227716 | PYROCATECHOL | C6 H6 O2 | 110.0366 |
| 11.17411897 | 10.76542254 | 1.037963807 | 0.141253657 | 0.45227716 | CYTIDINE MONOPHOSPHATE | C9 H14 N3 O8 P | 323.0519 |
| 16.3205964 | 15.8335683 | 1.030759213 | 0.141607545 | 0.45227716 | 1-METHYLADENOSINE | C11 H15 N5 O4 | 281.1132 |
| 19.02401875 | 18.2289535 | 1.043615518 | 0.146660819 | 0.463685217 | ADENOSINE | C10 H13 N5 O4 | 267.0971 |
| 15.61135267 | 16.05324825 | 0.972473136 | 0.149605347 | 0.46519303 | LYSINE | C6 H14 N2 O2 | 146.1053 |
| 20.58076827 | 20.06304724 | 1.025804706 | 0.150110211 | 0.46519303 | Hexanoylcarnitine | C13 H25 N O4 | 259.1789 |
| 14.75687447 | 14.31782901 | 1.030664248 | 0.152194714 | 0.467028877 | SALSOLINOL | C10 H13 N O2 | 179.0956 |
| 12.07236948 | 13.55482637 | 0.890632543 | 0.153828815 | 0.46746038 | PHENYLLACTATE | C9 H10 O3 | 166.0625 |
| 21.94038667 | 21.43622018 | 1.023519375 | 0.156665285 | 0.471502252 | AZELATE | C9 H16 O4 | 188.1058 |
| 19.44920307 | 19.10817255 | 1.017847364 | 0.159737755 | 0.473968003 | OXOPROLINE | C5 H7 N O3 | 129.0432 |
| 12.62901707 | 13.64839466 | 0.925311539 | 0.160513126 | 0.473968003 | L-ORNITHINE | C5 H12 N2 O2 | 132.0897 |
| 16.69113983 | 16.37220622 | 1.019480185 | 0.164727153 | 0.481865411 | BENZYLAMINE | C7 H9 N | 107.0737 |
| 11.50616555 | 11.1202719 | 1.034701818 | 0.17083872 | 0.495115919 | URACIL | C4 H4 N2 O2 | 112.0271 |
| 11.77208843 | 12.6020129 | 0.9341435 | 0.181131831 | 0.512990495 | 2,5-DIHYDROXYBENZOATE | C7 H6 O4 | 154.0266 |
| 15.52944266 | 15.28736691 | 1.015835019 | 0.181272139 | 0.512990495 | ADIPATE | C6 H10 O4 | 146.0581 |
| 13.05538653 | 13.72970711 | 0.950886018 | 0.181923147 | 0.512990495 | PALMITATE | C16 H32 O2 | 256.2399 |
| 9.517073283 | 8.865131988 | 1.073539942 | 0.184579676 | 0.515560627 | CDP-ETHANOLAMINE | C11 H20 N4 O11 P2 | 446.0607 |
| 16.99747671 | 16.56818403 | 1.025910666 | 0.186395214 | 0.515560627 | GUANINE | C5 H5 N5 O | 151.0505 |
| 16.15863464 | 18.4385188 | 0.876352098 | 0.187776075 | 0.515560627 | ASCORBATE | C6 H8 O6 | 176.0327 |
| 23.8488495 | 23.23635445 | 1.026359344 | 0.189930967 | 0.516942544 | CREATINE | C4 H9 N3 O2 | 131.0698 |
| 22.01001379 | 21.69710661 | 1.014421609 | 0.200966959 | 0.540315581 | 5-AMINOPENTANOATE | C5 H11 N O2 | 117.0796 |
| 12.74989235 | 12.51225457 | 1.018992403 | 0.202842128 | 0.540315581 | N-ACETYLASPARAGINE | C6 H10 N2 O4 | 174.0653 |
| 9.734058892 | 10.1676721 | 0.957353738 | 0.203697248 | 0.540315581 | 3,4-DIHYDROXYBENZOATE | C7 H6 O4 | 154.0267 |
| 8.625265444 | 8.250637125 | 1.045405987 | 0.206141309 | 0.542203612 | N-FORMYLGLYCINE | C3 H5 N O3 | 103.028 |
| 12.81652592 | 12.3068954 | 1.041410161 | 0.213168043 | 0.552998757 | SPERMINE | C10 H26 N4 | 202.2162 |
| 17.52376419 | 17.13208798 | 1.022862141 | 0.213779072 | 0.552998757 | SUBERATE | C8 H14 O4 | 174.0895 |
| 17.00394408 | 17.31565638 | 0.981998239 | 0.22208723 | 0.569781172 | GUANIDINOACETATE | C3 H7 N3 O2 | 117.0547 |
| 14.0551138 | 14.80338571 | 0.94945265 | 0.22731683 | 0.57845665 | DOCOSAHEXAENOATE | C22 H32 O2 | 328.2389 |
| 10.69381275 | 10.39977915 | 1.028273062 | 0.231750893 | 0.582190965 | LACTOSE | C12 H22 O11 | 342.1158 |
| 14.2549583 | 14.00713238 | 1.017692838 | 0.232504379 | 0.582190965 | N-ACETYLSERINE | C5 H9 N O4 | 147.0533 |
| 12.48286534 | 12.67090052 | 0.985160078 | 0.239367551 | 0.594619392 | SERINE | C3 H7 N O3 | 105.0429 |
| 16.57861873 | 16.38703368 | 1.011691259 | 0.249705118 | 0.614923209 | TRIMETHYLAMINE | C3 H9 N | 59.0732 |
| 13.66057646 | 14.00729569 | 0.97524724 | 0.252434907 | 0.614923209 | THIAMINE PYROPHOSPHATE | C12 H19 N4 O7 P2 S | 425.0488 |
| 9.192764613 | 8.838303295 | 1.040105132 | 0.253434805 | 0.614923209 | MANDELATE | C8 H8 O3 | 152.0474 |
| 16.14434078 | 15.88752105 | 1.016164871 | 0.25819763 | 0.621318616 | 3-HYDROXYBENZYL ALCOHOL | C7 H8 O2 | 124.0525 |
| 13.76972264 | 13.48406401 | 1.021184906 | 0.260040699 | 0.621318616 | NICOTINAMIDE MONONUCLEOTIDE | C11 H15 N2 O8 P | 334.0574 |
| 12.48351084 | 12.16173885 | 1.026457729 | 0.263215912 | 0.624140761 | PUTRESCINE-NH3 | C4 H9 N | 71.0731 |
| 12.63049179 | 12.22834056 | 1.03288682 | 0.277175729 | 0.650700785 | CYTOSINE | C4 H5 N3 O | 111.0434 |
| 23.61554478 | 23.29495634 | 1.013762139 | 0.280148587 | 0.650700785 | DEOXYCARNITINE | C7 H15 N O2 | 145.1106 |
| 16.22441034 | 16.56549362 | 0.979410014 | 0.280653693 | 0.650700785 | THIAMINE MONOPHOSPHATE | C12 H17 N4 O4 P S | 344.0667 |
| 15.52199222 | 15.08833127 | 1.028741478 | 0.286107175 | 0.651471735 | ASPARTATE | C4 H7 N O4 | 133.038 |
| 14.29191869 | 14.03280766 | 1.018464661 | 0.287686562 | 0.651471735 | NICOTINATE | C6 H5 N O2 | 123.0324 |
| 17.28901517 | 16.87243614 | 1.024689916 | 0.288978485 | 0.651471735 | ADENOSINE-MONOPHOSPHATE | C10 H14 N5 O7 P | 347.0644 |
| 11.73637948 | 12.04330724 | 0.974514662 | 0.290137427 | 0.651471735 | TRANS-ACONITATE | C6 H6 O6 | 174.0161 |
| 9.04530851 | 9.503033359 | 0.951833816 | 0.291393108 | 0.651471735 | CYCLIC AMP | C10 H12 N5 O6 P | 329.052 |
| 16.2063584 | 15.89248987 | 1.019749487 | 0.302920891 | 0.669689631 | DEOXYADENOSINE | C10 H13 N5 O3 | 251.1032 |
| 16.00731711 | 15.71923284 | 1.018326865 | 0.306727771 | 0.669689631 | 4-GUANIDINOBUTANOATE | C5 H11 N3 O2 | 145.0853 |
| 14.26063631 | 13.85378241 | 1.029367713 | 0.308206282 | 0.669689631 | HYDROXYKYNURENINE | C10 H12 N2 O4 | 224.08 |
| 17.03838263 | 16.70954188 | 1.019679818 | 0.314685065 | 0.669689631 | ADENINE | C5 H5 N5 | 135.0552 |
| 13.023508 | 14.12079599 | 0.922292767 | 0.317181432 | 0.669689631 | Chenodeoxycholic acid | C24 H40 O4 | 392.2909 |
| 12.91799507 | 12.58741056 | 1.026263107 | 0.318319677 | 0.669689631 | L-ALANINE | C3 H7 N O2 | 89.0481 |
| 14.85768312 | 14.57710767 | 1.019247676 | 0.318513538 | 0.669689631 | 6-HYDROXYDOPAMINE | C8 H11 N O3 | 169.0743 |
| 12.74029411 | 12.53705007 | 1.016211473 | 0.319100093 | 0.669689631 | NAD+ | C21 H27 N7 O14 P2 | 663.1092 |
| 13.55867715 | 13.19583338 | 1.027496844 | 0.320170751 | 0.669689631 | N-ACETYLMETHIONINE | C7 H13 N O3 S | 191.0614 |
| 15.69632618 | 15.46599127 | 1.014892994 | 0.320937523 | 0.669689631 | 3-METHOXYTYRAMINE | C9 H13 N O2 | 167.0946 |
| 15.54046701 | 15.14748741 | 1.02594355 | 0.330378442 | 0.68130497 | PANTOTHENATE | C9 H17 N O5 | 219.1106 |
| 16.28050763 | 16.54438232 | 0.984050496 | 0.332375583 | 0.68130497 | URIDINE | C9 H12 N2 O6 | 244.0699 |
| 18.09878116 | 17.83656981 | 1.014700772 | 0.333034059 | 0.68130497 | PHOSPHORYLCHOLINE | C5 H14 N O4 P | 183.066 |
| 17.06256816 | 16.49166012 | 1.034617985 | 0.339492354 | 0.690007187 | Adipoyl-L-carnitine | C13 H23 N O6 | 289.1527 |
| 18.47249596 | 17.00371705 | 1.086379872 | 0.342023225 | 0.690666255 | INOSINE | C10 H12 N4 O5 | 268.082 |
| 20.13323785 | 19.82629537 | 1.015481585 | 0.348086956 | 0.698405239 | O-ACETYLCARNITINE | C9 H17 N O4 | 203.1171 |
| 19.78376122 | 19.99465995 | 0.989452247 | 0.351515974 | 0.700792994 | 3-METHYL-2-OXOVALERATE | C6 H10 O3 | 130.0634 |
| 10.19855556 | 9.751841646 | 1.04580816 | 0.354197614 | 0.701669956 | BUTANOATE | C4 H8 O2 | 88.0524 |
| 19.63348743 | 19.40670294 | 1.011685885 | 0.358756074 | 0.706230511 | TAURINE | C2 H7 N O3 S | 125.0157 |
| 19.22027597 | 18.84890526 | 1.019702508 | 0.363663301 | 0.711416332 | STEAROYLCARNITINE | C25 H49 N O4 | 427.3649 |
| 13.67977603 | 13.38968495 | 1.021665265 | 0.367017363 | 0.712751495 | THYROXINE | C15 H11 I4 N O4 | 776.6822 |
| 23.90146072 | 23.7585531 | 1.006014997 | 0.371145133 | 0.712751495 | URATE | C5 H4 N4 O3 | 168.0293 |
| 18.68060809 | 18.44626157 | 1.012704283 | 0.37488599 | 0.712751495 | 2-HYDROXYPYRIDINE | C5 H5 N O | 95.0368 |
| 12.70603495 | 12.09793117 | 1.050265105 | 0.375308648 | 0.712751495 | Tauro-?-muricholic acid | C26 H45 N O7 S | 515.2881 |
| 10.9811811 | 9.94174606 | 1.104552564 | 0.376179524 | 0.712751495 | Ursodeoxycholic acid | C24 H40 O4 | 392.2913 |
| 21.90275827 | 21.49881555 | 1.018789069 | 0.37800878 | 0.712751495 | LPE(18:1) | C23 H46 N O7 P | 479.2996 |
| 13.18384489 | 12.9724635 | 1.016294622 | 0.38502326 | 0.720268804 | 3-(2-HYDROXYPHENYL)PROPANOATE | C9 H10 O3 | 166.0626 |
| 12.10817839 | 11.81123323 | 1.025140911 | 0.386597952 | 0.720268804 | SORBITOL | C6 H14 O6 | 182.0787 |
| 7.854262755 | 8.010421834 | 0.980505511 | 0.407682228 | 0.733719528 | THYMINE | C5 H6 N2 O2 | 126.0425 |
| 10.77919 | 10.46439321 | 1.030082661 | 0.408640623 | 0.733719528 | 3-HYDROXYMETHYLGLUTARATE | C6 H10 O5 | 162.0527 |
| 13.68103498 | 13.50570482 | 1.012981934 | 0.409806972 | 0.733719528 | N-FORMYL-L-METHIONINE | C6 H11 N O3 S | 177.0457 |
| 15.50960631 | 15.28881331 | 1.014441474 | 0.412551502 | 0.733719528 | N-ACETYLPUTRESCINE | C6 H14 N2 O | 130.1107 |
| 10.68265304 | 10.43373952 | 1.023856597 | 0.413269402 | 0.733719528 | N1-ACETYLSPERMINE | C12 H28 N4 O | 244.2264 |
| 9.971172255 | 10.37970826 | 0.960640897 | 0.414706645 | 0.733719528 | PYRUVATE | C3 H4 O3 | 88.016 |
| 13.28808324 | 13.09918802 | 1.014420376 | 0.416174041 | 0.733719528 | AMINOADIPATE | C6 H11 N O4 | 161.069 |
| 14.02986394 | 13.76930752 | 1.018922987 | 0.416406041 | 0.733719528 | 6-HYDROXYNICOTINATE | C6 H5 N O3 | 139.0269 |
| 8.426144564 | 8.277403846 | 1.017969489 | 0.417140228 | 0.733719528 | XANTHURENATE | C10 H7 N O4 | 205.0356 |
| 8.933950539 | 9.470352567 | 0.943359867 | 0.418818313 | 0.733719528 | (3ALPHA,5BETA,7ALPHA)-3,7-DIHYDROXY-12-OXOCHOLAN-24-OIC ACID | C24 H38 O5 | 406.2703 |
| 18.93026386 | 18.67224739 | 1.01381818 | 0.41960318 | 0.733719528 | CITRULLINE | C6 H13 N3 O3 | 175.0958 |
| 14.8317621 | 14.55864957 | 1.018759469 | 0.422581604 | 0.734822457 | N-ACETYLASPARTYLGLUTAMATE | C11 H16 N2 O8 | 304.0907 |
| 20.22500062 | 20.03487747 | 1.009489609 | 0.430030712 | 0.737833994 | TYROSINE | C9 H11 N O3 | 181.0746 |
| 12.07023836 | 12.25133966 | 0.985217837 | 0.431116109 | 0.737833994 | 2-Hydroxytetradecanoic acid | C14 H28 O3 | 244.2035 |
| 7.538330841 | 7.3214795 | 1.029618514 | 0.43138537 | 0.737833994 | EPINEPHRINE | C9 H13 N O3 | 183.0894 |
| 13.13112637 | 12.939773 | 1.014788001 | 0.440484583 | 0.742864355 | HYDROXYISOCAPROIC | C6 H12 O3 | 132.0786 |
| 8.794215735 | 8.601792479 | 1.022370135 | 0.442486332 | 0.742864355 | DEOXYCYTIDINE | C9 H13 N3 O4 | 227.0909 |
| 13.12218106 | 12.93131169 | 1.014760248 | 0.445510347 | 0.742864355 | 2-Hydroxyhexanoic acid | C6 H12 O3 | 132.0786 |
| 17.04338183 | 16.82760411 | 1.012822843 | 0.448651018 | 0.742864355 | HYDROXYPHENYLLACTATE | C9 H10 O4 | 182.0579 |
| 17.5372005 | 17.24331334 | 1.017043543 | 0.448935537 | 0.742864355 | DL-2-AMINOCTANOATE | C8 H17 N O2 | 159.1257 |
| 8.251561386 | 8.480915695 | 0.972956422 | 0.452882474 | 0.742864355 | BETA-NICOTINAMIDE ADENINE DINUCLEOTIDE | C21 H29 N7 O14 P2 | 665.1235 |
| 9.607218373 | 9.453468208 | 1.01626389 | 0.454145395 | 0.742864355 | 5-Aminopentanoic acid | C5 H11 N O2 | 117.0791 |
| 9.924520884 | 10.08347842 | 0.984235843 | 0.454830794 | 0.742864355 | GLUTATHIONE REDUCED | C10 H17 N3 O6 S | 307.0844 |
| 10.15587487 | 10.32708296 | 0.983421447 | 0.456459521 | 0.742864355 | GLYCERALDEHYDE 3-PHOSPHATE | C3 H7 O6 P | 169.9973 |
| 12.1130746 | 11.87544142 | 1.020010472 | 0.460854431 | 0.742864355 | N-ACETYLGLYCINE | C4 H7 N O3 | 117.0432 |
| 10.63747368 | 10.29410032 | 1.033356325 | 0.463013102 | 0.742864355 | MANNITOL | C6 H14 O6 | 182.0794 |
| 14.05357673 | 13.33022291 | 1.054264195 | 0.469754598 | 0.742864355 | DEOXYINOSINE | C10 H11 N4 Na O4 | 274.068 |
| 11.68611818 | 11.48154872 | 1.017817235 | 0.472078679 | 0.742864355 | 3-METHOXYTYROSINE | C10 H13 N O4 | 211.0842 |
| 8.582674533 | 8.821689462 | 0.972905992 | 0.472534165 | 0.742864355 | DEOXYADENOSINE MONOPHOSPHATE | C10 H14 N5 O6 P | 331.0678 |
| 13.04716935 | 13.18193811 | 0.989776256 | 0.472585753 | 0.742864355 | VALINE D8 | C5 H3 D8 N O2 | 125.1298 |
| 14.69692914 | 14.44311302 | 1.017573505 | 0.474575505 | 0.742864355 | N,N,N-TRIMETHYLLYSINE | C9 H20 N2 O2 | 188.1526 |
| 20.42511093 | 20.31985027 | 1.005180189 | 0.47467371 | 0.742864355 | TRYPTOPHAN D5 | C11 H7 D5 N2 O2 | 209.1222 |
| 12.87432123 | 13.03515518 | 0.987661524 | 0.486776193 | 0.754855773 | QUINOLINATE | C7 H5 N O4 | 167.0203 |
| 15.80850475 | 15.65904654 | 1.009544528 | 0.487159317 | 0.754855773 | BENZOATE | C7 H6 O2 | 122.037 |
| 7.235499589 | 7.066204341 | 1.023958442 | 0.492605141 | 0.756884228 | Lactoylglutathione | C13 H21 N3 O8 S | 379.1044 |
| 17.96345587 | 17.67285948 | 1.016443088 | 0.493475376 | 0.756884228 | 1-METHYL-L-HISTIDINE | C7 H11 N3 O2 | 169.0851 |
| 11.72734344 | 11.84025512 | 0.990463747 | 0.496605904 | 0.756884228 | CITRAMALATE | C5 H8 O5 | 148.037 |
| 24.64207945 | 24.45267374 | 1.007745807 | 0.498141058 | 0.756884228 | CREATININE | C4 H7 N3 O | 113.0591 |
| 13.73319076 | 13.56480545 | 1.012413396 | 0.506457653 | 0.765803118 | MALEIMIDE | C4 H3 N O2 | 97.0167 |
| 27.5672338 | 27.41629128 | 1.005505578 | 0.513071907 | 0.772074553 | LPC(16:0) | C24 H50 N O7 P | 495.3316 |
| 21.20818963 | 20.92503852 | 1.013531689 | 0.518588973 | 0.776642816 | PIPECOLATE | C6 H11 N O2 | 129.0797 |
| 25.74454348 | 25.60813842 | 1.005326629 | 0.525072998 | 0.782608801 | BETAINE | C5 H11 N O2 | 117.0793 |
| 10.85160046 | 10.65555586 | 1.018398345 | 0.531594981 | 0.788574545 | 7-METHYLGUANOSINE | C11 H15 N5 O5 | 297.107 |
| 16.9265849 | 16.65189717 | 1.016495882 | 0.535354524 | 0.7904055 | trans-2-Octenoyl-L-carnitine | C15 H27 N O4 | 285.1938 |
| 14.60365604 | 14.45828483 | 1.010054526 | 0.538149859 | 0.790802375 | SARCOSINE | C3 H7 N O2 | 89.0474 |
| 15.40862884 | 15.79970007 | 0.975248187 | 0.544339718 | 0.796160429 | OLEATE | C18 H34 O2 | 282.2556 |
| 12.38936432 | 12.7064057 | 0.975048697 | 0.554352133 | 0.804028054 | HOMOCYSTEINE | C4 H9 N O2 S | 135.037 |
| 10.40667986 | 10.30338418 | 1.010025414 | 0.554856421 | 0.804028054 | DEOXYGUANOSINE | C10 H13 N5 O4 | 267.0965 |
| 14.38799074 | 14.57100258 | 0.987439997 | 0.562753989 | 0.806182866 | HISTIDINE | C6 H9 N3 O2 | 155.07 |
| 19.08524164 | 18.93435869 | 1.007968738 | 0.566519282 | 0.806182866 | THREONINE | C4 H9 N O3 | 119.0581 |
| 12.07931893 | 11.95040161 | 1.010787697 | 0.567289813 | 0.806182866 | 2-HYDROXY-4-(METHYLTHIO)BUTANOATE | C5 H10 O3 S | 150.0347 |
| 20.51169983 | 20.31225997 | 1.009818694 | 0.569156868 | 0.806182866 | LPE(16:0) | C21 H44 N O7 P | 453.2851 |
| 14.48282274 | 14.29109429 | 1.013415939 | 0.571541128 | 0.806182866 | PANTOLACTONE | C6 H10 O3 | 130.0615 |
| 14.28055953 | 14.06162996 | 1.015569288 | 0.574164546 | 0.806182866 | 3-Hydroxyoctanoic acid | C8 H16 O3 | 160.1098 |
| 14.05358111 | 13.87230555 | 1.013067443 | 0.574373096 | 0.806182866 | HYPOTAURINE | C2 H7 N O2 S | 109.0211 |
| 16.73352755 | 16.54621754 | 1.011320413 | 0.582075408 | 0.807657206 | PYRIDOXAL | C8 H9 N O3 | 167.0566 |
| 16.01593387 | 15.78156031 | 1.014851102 | 0.58282484 | 0.807657206 | S-ADENOSYLMETHIONINE | C15 H22 N6 O5 S | 398.1368 |
| 12.20129393 | 12.65705681 | 0.963991401 | 0.583164628 | 0.807657206 | URSODEOXYCHOLATE | C24 H40 O4 | 392.2912 |
| 17.648346 | 17.18557793 | 1.026927698 | 0.588747592 | 0.81179734 | 8-HYDROXY-DEOXYGUANOSINE | C10 H13 N5 O5 | 283.0921 |
| 14.70790891 | 14.84670197 | 0.99065159 | 0.601313572 | 0.823653943 | 2-QUINOLINECARBOXYLATE | C10 H7 N O2 | 173.0479 |
| 9.628173403 | 9.739729947 | 0.988546238 | 0.603319868 | 0.823653943 | ETHANOLAMINE PHOSPHATE | C2 H8 N O4 P | 141.02 |
| 8.451007097 | 8.297867766 | 1.018455263 | 0.605240917 | 0.823653943 | ANSERINE | C10 H16 N4 O3 | 240.1231 |
| 15.58702673 | 15.46582187 | 1.007836949 | 0.609312748 | 0.825589976 | N-METHYLGLUTAMATE | C6 H11 N O4 | 161.069 |
| 10.82586886 | 11.10932169 | 0.974485136 | 0.614535096 | 0.825589976 | GAMMA-LINOLENATE | C18 H30 O2 | 278.224 |
| 16.54031247 | 16.3387044 | 1.012339294 | 0.614576564 | 0.825589976 | GLUCOSAMINE | C6 H13 N O5 | 179.0789 |
| 14.29367944 | 14.20990531 | 1.005895474 | 0.621917621 | 0.828654821 | OMEGA-HYDROXYDODECANOATE | C12 H24 O3 | 216.1725 |
| 10.76084086 | 10.66893384 | 1.008614452 | 0.622152981 | 0.828654821 | FUMARATE | C4 H4 O4 | 116.0107 |
| 11.96962787 | 12.09828769 | 0.989365452 | 0.626459882 | 0.830855691 | 10-Undecenoic acid | C11 H20 O2 | 184.1464 |
| 12.58170182 | 12.99349147 | 0.968308006 | 0.630415384 | 0.832573904 | Murideoxycholic Acid | C24 H40 O4 | 392.2916 |
| 16.13036058 | 16.59081127 | 0.972246644 | 0.638465408 | 0.838671133 | CORTEXOLONE | C21 H30 O4 | 346.2135 |
| 12.53575748 | 12.60965345 | 0.99413973 | 0.643397274 | 0.838671133 | N-ACETYLASPARTATE | C6 H9 N O5 | 175.0484 |
| 11.63059403 | 11.76607896 | 0.988485125 | 0.64524998 | 0.838671133 | HOMOVANILLATE | C9 H10 O4 | 182.0565 |
| 11.93560347 | 12.04538758 | 0.990885797 | 0.645749978 | 0.838671133 | GLUCONOLACTONE | C6 H10 O6 | 178.0473 |
| 15.28068419 | 14.95307128 | 1.021909406 | 0.662697285 | 0.857125001 | SEROTONIN | C10 H12 N2 O | 176.0951 |
| 11.3814209 | 11.23072244 | 1.013418412 | 0.6774839 | 0.870531843 | Fructose 1,6-bisphosphate | C6 H14 O12 P2 | 339.9951 |
| 15.28382692 | 15.34467068 | 0.99603486 | 0.678625462 | 0.870531843 | ASPARTATE D3 | C4 H4 D3 N O4 | 136.0567 |
| 17.58923248 | 17.14415121 | 1.025961114 | 0.692285348 | 0.881299618 | Glycodeoxycholic acid | C26 H43 N O5 | 449.3132 |
| 15.11395598 | 15.22812106 | 0.992503009 | 0.693480923 | 0.881299618 | AICAR | C9 H15 N4 O8 P | 338.0602 |
| 19.1180506 | 19.02061699 | 1.005122526 | 0.695466472 | 0.881299618 | BETAINE ALDEHYDE | C5 H11 N O | 101.084 |
| 12.18535967 | 12.09026667 | 1.007865253 | 0.699353382 | 0.882651648 | 4-ACETAMIDOBUTANOATE | C6 H11 N O3 | 145.0737 |
| 14.19709932 | 14.07800032 | 1.008459937 | 0.711097886 | 0.892510753 | GUANIDINOSUCCINATE | C5 H9 N3 O4 | 175.0593 |
| 16.46645443 | 16.38248955 | 1.005125282 | 0.714975109 | 0.892510753 | DIETHANOLAMINE | C4 H11 N O2 | 105.0787 |
| 21.30759041 | 21.16065975 | 1.006943577 | 0.725174406 | 0.892510753 | octanoylcarnitine | C15 H29 N O4 | 287.21 |
| 11.12085743 | 11.27524064 | 0.986307768 | 0.725230062 | 0.892510753 | ACETOACETATE | C4 H6 O3 | 102.0315 |
| 14.54044598 | 14.74050333 | 0.986428051 | 0.725244116 | 0.892510753 | LINOLEATE | C18 H32 O2 | 280.2399 |
| 21.02320732 | 20.96062773 | 1.002985578 | 0.726798862 | 0.892510753 | GLUTAMINE | C5 H10 N2 O3 | 146.0704 |
| 23.60578374 | 23.51084388 | 1.00403813 | 0.729597249 | 0.892510753 | GLYCEROPHOSPHOCHOLINE | C8 H20 N O6 P | 257.1032 |
| 12.42309883 | 12.67207704 | 0.980352218 | 0.729976846 | 0.892510753 | Phenylacetic acid | C8 H8 O2 | 136.052 |
| 16.45175401 | 16.53122154 | 0.995192882 | 0.733286935 | 0.893069302 | ALPHA-KETOISOVALERATE | C5 H8 O3 | 116.0473 |
| 13.51173705 | 13.40849678 | 1.007699615 | 0.761873376 | 0.924288243 | HISTAMINE | C5 H9 N3 | 111.0796 |
| 13.38394204 | 13.47916811 | 0.992935315 | 0.765245 | 0.924794151 | Traumatic acid | C12 H20 O4 | 228.1358 |
| 10.30865491 | 10.4057134 | 0.990672577 | 0.770983177 | 0.928145133 | STACHYOSE | C24 H42 O21 | 666.219 |
| 19.8058678 | 19.94648079 | 0.992950486 | 0.776208307 | 0.929780854 | N-ACETYLORNITHINE | C7 H14 N2 O3 | 174.1011 |
| 10.13760591 | 10.05659997 | 1.008055002 | 0.778283015 | 0.929780854 | CYTIDINE 2',3'-CYCLIC PHOSPHATE | C9 H12 N3 O7 P | 305.041 |
| 13.24714446 | 13.19537668 | 1.003923176 | 0.782008801 | 0.930679675 | FAD | C27 H33 N9 O15 P2 | 785.1535 |
| 14.59360617 | 14.54906282 | 1.003061596 | 0.789474931 | 0.931022771 | GLUTARATE | C5 H8 O4 | 132.0422 |
| 15.07780381 | 15.02892146 | 1.003252552 | 0.79063419 | 0.931022771 | UREA | C H4 N2 O | 60.0321 |
| 12.30440095 | 12.37109191 | 0.994609128 | 0.79122063 | 0.931022771 | 4-QUINOLINECARBOXYLATE | C10 H7 N O2 | 173.0478 |
| 12.27395765 | 12.35539526 | 0.993408741 | 0.797196089 | 0.934540734 | 3-METHYLHISTAMINE | C6 H11 N3 | 125.095 |
| 8.366687861 | 8.29851659 | 1.008214874 | 0.812362835 | 0.939812298 | FERULATE | C10 H10 O4 | 194.059 |
| 16.29245228 | 16.62249012 | 0.980145102 | 0.814777075 | 0.939812298 | Glycoursodeoxycholic acid | C26 H43 N O5 | 449.3132 |
| 11.81393646 | 11.85268474 | 0.996730843 | 0.818876868 | 0.939812298 | DEOXYGUANOSINE-MONOPHOSPHATE | C10 H14 N5 O7 P | 347.0642 |
| 12.88728334 | 12.66694888 | 1.017394439 | 0.820154979 | 0.939812298 | GUANOSINE | C10 H13 N5 O5 | 283.0923 |
| 6.918056056 | 6.825865108 | 1.013506119 | 0.82133141 | 0.939812298 | CITICOLINE | C14 H26 N4 O11 P2 | 488.1056 |
| 8.430115811 | 8.33250955 | 1.011713909 | 0.821455804 | 0.939812298 | OPHTHALMATE | C11 H19 N3 O6 | 289.1272 |
| 11.30869335 | 11.44349994 | 0.988219812 | 0.822711086 | 0.939812298 | EICOSAPENTAENOATE | C20 H30 O2 | 302.2236 |
| 14.26677762 | 14.21211254 | 1.003846373 | 0.827629529 | 0.941992882 | HOMOSERINE | C4 H9 N O3 | 119.0584 |
| 26.47437297 | 26.43237023 | 1.001589064 | 0.839836894 | 0.950431148 | L-CARNITINE | C7 H15 N O3 | 161.1057 |
| 16.32393137 | 16.60193952 | 0.983254478 | 0.84415021 | 0.950431148 | Glycohyodeoxycholic Acid | C26 H43 N O5 | 449.3131 |
| 14.01193673 | 13.8801017 | 1.009498132 | 0.844152904 | 0.950431148 | CHOLATE | C24 H40 O5 | 408.2862 |
| 17.44250449 | 17.26987638 | 1.009995909 | 0.855237254 | 0.954650238 | GLYCOCHOLATE | C26 H43 N O6 | 465.308 |
| 27.65767679 | 27.61199 | 1.0016546 | 0.855644095 | 0.954650238 | LPC(18:1) | C26 H52 N O7 P | 521.3464 |
| 13.41877878 | 13.34933837 | 1.005201787 | 0.857513368 | 0.954650238 | GLYCEROL 3-PHOSPHATE | C3 H9 O6 P | 172.0142 |
| 10.13402163 | 10.17812878 | 0.995666477 | 0.860100214 | 0.954650238 | GUANOSINE MONOPHOSPHATE | C10 H14 N5 O8 P | 363.0575 |
| 12.76393155 | 12.70709584 | 1.004472754 | 0.864408242 | 0.956041625 | 2-Ethyl-2-hydroxybutyric acid | C6 H12 O3 | 132.0786 |
| 13.37105278 | 13.33158118 | 1.002960759 | 0.868069178 | 0.956710044 | 2-Hydroxyhexadecanoic acid | C16 H32 O3 | 272.2349 |
| 11.69216326 | 11.76376801 | 0.993913111 | 0.876326898 | 0.960179079 | 3-SULFINOALANINE | C3 H7 N O4 S | 153.0097 |
| 7.776877132 | 7.822601327 | 0.994154861 | 0.87735213 | 0.960179079 | CARNOSINE | C9 H14 N4 O3 | 226.1074 |
| 13.18760436 | 13.15478896 | 1.002494559 | 0.884096514 | 0.963374816 | NORMETANEPHRINE | C9 H13 N O3 | 183.0902 |
| 17.17178673 | 17.11696104 | 1.003203004 | 0.886427945 | 0.963374816 | UROCANATE | C6 H6 N2 O2 | 138.0438 |
| 9.810577403 | 9.848659129 | 0.996133308 | 0.893082955 | 0.965931143 | 3-METHYL-2-OXINDOLE | C9 H9 N O | 147.0686 |
| 12.93479981 | 12.96035584 | 0.998028138 | 0.894952177 | 0.965931143 | 7-HYDROXYOCTANOATE | C8 H16 O3 | 160.1099 |
| 11.19034379 | 11.16553282 | 1.002222103 | 0.914998832 | 0.970063292 | ACETOIN | C4 H8 O2 | 88.0522 |
| 19.98180359 | 19.95753671 | 1.001215926 | 0.915547883 | 0.970063292 | N,N-DIMETHYLARGININE | C8 H18 N4 O2 | 202.1432 |
| 9.666231755 | 9.644050323 | 1.002300012 | 0.918069103 | 0.970063292 | OXOADIPATE | C6 H8 O5 | 160.0379 |
| 12.8265272 | 12.80445782 | 1.00172357 | 0.920860483 | 0.970063292 | HEXOSE | C6 H12 O6 | 180.0641 |
| 10.04277618 | 10.10789097 | 0.993558024 | 0.92389807 | 0.970063292 | Muricholic Acid | C24 H40 O5 | 408.286 |
| 14.54600112 | 14.51630863 | 1.002045457 | 0.924122242 | 0.970063292 | 4-HYDROXY-3-METHOXYPHENYLGLYCOL | C9 H12 O4 | 184.0718 |
| 9.703076585 | 9.686127373 | 1.001749844 | 0.926720085 | 0.970063292 | INOSINE MONOPHOSPHATE | C10 H13 N4 O8 P | 348.0477 |
| 11.73595843 | 11.76254513 | 0.997739715 | 0.93151965 | 0.970063292 | ALLANTOIN | C4 H6 N4 O3 | 158.0439 |
| 10.07386627 | 10.04576178 | 1.002797646 | 0.936422508 | 0.970063292 | URIDINE MONOPHOSPHATE | C9 H13 N2 O9 P | 324.0361 |
| 13.92094016 | 13.90248905 | 1.001327181 | 0.936813368 | 0.970063292 | BENZYL ALCOHOL | C7 H8 O | 108.0576 |
| 19.10252972 | 19.12418339 | 0.998867733 | 0.938089776 | 0.970063292 | 2-HYDROXYBUTYRATE | C4 H8 O3 | 104.0476 |
| 10.34076739 | 10.3604414 | 0.998101046 | 0.938579524 | 0.970063292 | GLYCOLATE | C2 H4 O3 | 76.0164 |
| 14.13452477 | 14.16320092 | 0.997975306 | 0.939239444 | 0.970063292 | 4-IMIDAZOLEACETATE | C5 H6 N2 O2 | 126.0418 |
| 18.70129954 | 18.716494 | 0.999188178 | 0.942170098 | 0.970063292 | 2-KETOHEXANOIC | C6 H10 O3 | 130.0633 |
| 15.71365413 | 15.72570746 | 0.999233527 | 0.953138904 | 0.97417463 | VALINE | C5 H11 N O2 | 117.0797 |
| 11.31105657 | 11.31962744 | 0.999242831 | 0.953757788 | 0.97417463 | METHYL VANILLATE | C9 H10 O4 | 182.0574 |
| 13.07883295 | 13.05586958 | 1.001758854 | 0.955500356 | 0.97417463 | 4-PYRIDOXATE | C8 H9 N O4 | 183.0529 |
| 16.13689849 | 16.17192987 | 0.997833816 | 0.963429652 | 0.976738675 | Deoxycholic acid | C24 H40 O4 | 392.2917 |
| 16.13744833 | 16.17169102 | 0.997882554 | 0.964256391 | 0.976738675 | DEOXYCHOLATE | C24 H40 O4 | 392.2917 |
| 16.91891393 | 16.92367783 | 0.999718507 | 0.970617665 | 0.980010739 | GLUTAMATE D3 | C5 H6 D3 N O4 | 150.0726 |
| 12.6766875 | 12.67248534 | 1.000331597 | 0.976885478 | 0.98028452 | 10-HYDROXYDECANOATE | C10 H20 O3 | 188.1413 |
| 21.65237526 | 21.64831843 | 1.000187397 | 0.97715262 | 0.98028452 | TRYPTOPHAN | C11 H12 N2 O2 | 204.0909 |
| 19.55717008 | 19.54721021 | 1.000509529 | 0.992733134 | 0.992733134 | GLYCOCHENODEOXYCHOLATE | C26 H43 N O5 | 449.314 |

**Supplementary Table 4. Correlation between CLASI-A Scores vs. Metabolites in CLE Serum and Skin.**

| **Compound** | **Serum or Skin^a^** | **Spearman's rho** | **P-value** |
| --- | --- | --- | --- |
| Cystine | Serum | 0.44 | 0.24 |
| Arabinose | Serum | 0.7 | 0.04 |
| Citrulline | Skin | 0 | >0.99 |
| Uracil | Skin | 0.4 | 0.75 |
| N-acetylserine | Skin | 1 | 0.08 |
| Thiamine pyrophosphate | Skin | -0.8 | 0.33 |
| cADPr | Skin | -0.2 | 0.92 |
| NMN | Skin | 0.8 | 0.33 |
| NAD^+^ | Skin | 0.8 | 0.33 |
| 4-pyridoxate | Skin | 1 | 0.08 |
| Hippurate | Skin | 0.4 | 0.75 |
| Pipecolate | Skin | 0.2 | 0.92 |
| Quinate | Skin | 0.4 | 0.75 |

Abbreviations: cADPr: cyclic-ADP ribose; CLASI-A: Cutaneous Lupus Erythematosus Disease Area and Severity Index; NAD: nicotinamide adenine dinucleotide; NMN: nicotinamide mononucleotide.

^a^One skin sample was missing CLASI-A score and did not factor into the correlation analysis.
